# HySimODE: a hybrid stochastic-deterministic simulation framework for multiscale models of biological systems

**DOI:** 10.1093/bioinformatics/btag185

**Published:** 2026-04-17

**Authors:** Criseida G Zamora-Chimal, Alexander P S Darlington

**Affiliations:** School of Engineering, University of Warwick, Coventry, CV4 7AL, United Kingdom; School of Engineering, University of Warwick, Coventry, CV4 7AL, United Kingdom

## Abstract

Hybrid simulation is essential for modeling biochemical systems that mix low-copy stochastic dynamics with high-abundance deterministic processes. We present **HySimODE**, a Python framework that automates hybrid simulation directly from user-defined ordinary differential equation-based models. HySimODE uses a short deterministic pre-simulation and a machine-learning classifier to automatically assign each species to a stochastic or deterministic regime, and then combines a simple stochastic update rule with a stiff ODE solver in a single simulation loop. The classifier was trained and validated on a diverse dataset of biochemical ODE models spanning multiple dynamical regimes, enabling robust stochastic–deterministic partitioning beyond simple abundance thresholds. This design eliminates manual specification of regimes, avoids model reformulation, and enables reproducible, data-driven hybrid simulations of ODE-only biochemical models, including systems with saturable kinetics, effective-rate laws, or macro-energetic variables that lack a consistent reaction-network representation. Benchmarking against deterministic integrators, stochastic simulations, and abundance-threshold hybrid approaches demonstrates that HySimODE provides a practical and scalable framework for hybrid simulation of ODE-defined biochemical systems. We demonstrate its utility on two distinct case studies: a host–circuit interaction model from synthetic biology and a long-term synaptic potentiation model from neurobiology. HySimODE includes a modular adapter system that automatically converts ODE models written in concentrations into molecular counts, enabling universal compatibility across biochemical systems without code modification.

## Graphical Abstract

**Graphical Abstract:**
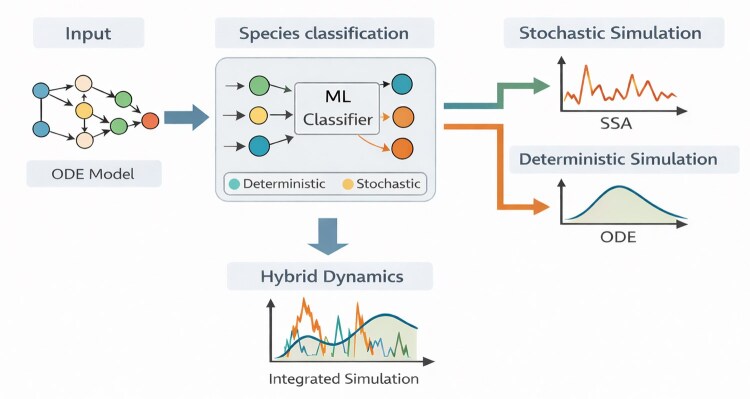

Key Messages

- HySimODE performs hybrid stochastic–deterministic simulation directly from ODE models.
- A machine learning classifier automatically partitions species into stochastic and deterministic regimes.
- No reaction-network reconstruction or manual threshold tuning is required.
- The framework generalizes across multiscale biochemical systems with diverse dynamics.

## Introduction

Biological models often span regimes where some molecular species are well approximated by continuous concentrations, while others require a discrete stochastic description due to low copy numbers or bursty production (Gillespie 1977, Elowitz et al. 2002). Hybrid solvers combine stochastic processes with ordinary differential equations (ODEs) to capture both behaviours efficiently (Haseltine and Rawlings 2002, Cao et al. 2005). However, a persistent challenge is the *partitioning problem*, i.e., deciding which species should be simulated stochastically and which deterministically. Strategies based on fixed abundance thresholds or model-specific heuristics are often brittle across systems and difficult to generalize to complex multiscale models. Existing hybrid approaches, such as that developed by Haseltine and Rawlings (Haseltine and Rawlings 2002), separate reactions or species into deterministic and stochastic subsets using heuristic rules or timescale criteria. While effective in some contexts, these methods typically require manual inspection of models, reimplementation of reaction networks, or extensive model-specific tuning.

Many widely used biological models are formulated directly as ODE systems using effective rate laws or coarse-grained variables, making reaction-network reconstruction ambiguous or impractical. Other approaches utilise timescale separation (e.g., Haseltine–Rawlings) to classify reactions using rate-based analytical criteria, while adaptive variance indicators or moment-based hybrid solvers couple ODE integration with stochastic approximations. Although effective when a mechanistic reaction network is available, these strategies cannot be directly applied to ODE-defined biochemical models that employ effective rates or coarse-grained mechanisms. Here we address this gap by developing methods that operate directly on the ODE formulation.

**Figure 1:**
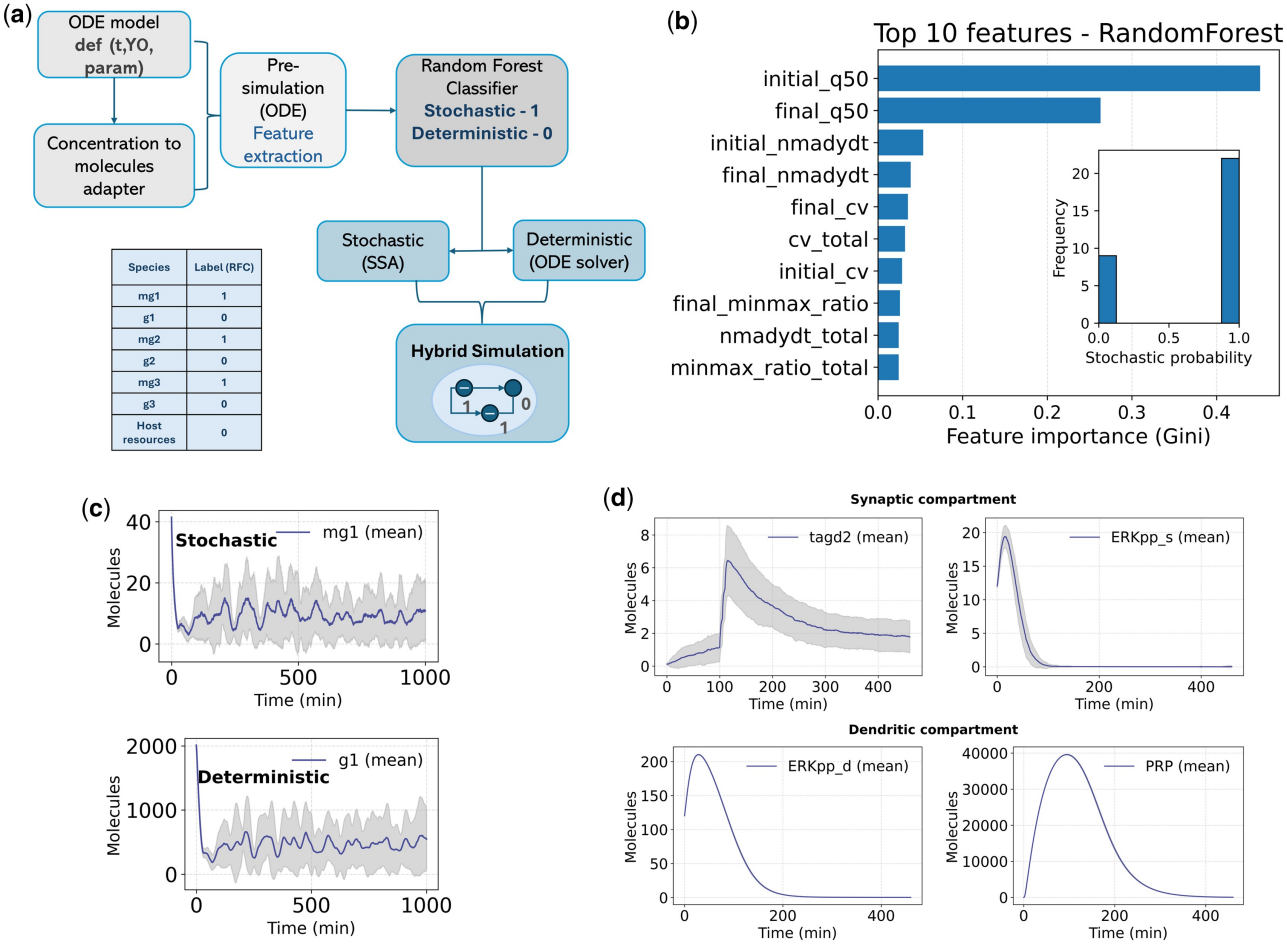
**Overview of HySimODE and validation on benchmark models**. (**a**) Workflow of HySimODE. An ODE model provides species, parameters, and initial conditions, which are optionally converted from concentrations to molecule counts. A deterministic pre-simulation is performed to extract statistical and dynamical features, which are classified by a pre-trained Random Forest into stochastic (1) or deterministic 0 regimes. The hybrid simulator integrates both domains concurrently, using event-driven stochastic updates for low-copy species and deterministic ODE solvers for high-copy species. (**b**) Feature importance analysis of the RFC. The classifier relies on a combination of trajectory magnitude and variability descriptors derived from deterministic simulations. The most important features indicate that the RFC captures nonlinear combinations of dynamical signatures rather than relying on a single abundance threshold. Inset: distribution of predicted stochastic probabilities across all species in the host-repressilator system. (**c**) Validation on the host–repressilator system (31 ODEs). The RFC assigns the mRNA mg1 to the *stochastic* regime (top) and the corresponding protein g1 to the *deterministic* regime (bottom). Mean trajectories and ±1 SD across repeated hybrid runs illustrate larger variability for the stochastic species and smoother dynamics for the deterministic species. (**d**) Validation on the Smolen synaptic tagging model (23 ODEs). Representative synaptic (tagd2, ERKpp_s) and dendritic (ERKpp_d, PRP) variables exhibit characteristic hybrid dynamics within the same simulation framework. Low-copy intermediates display stochastic fluctuations, whereas abundant signaling components follow smooth deterministic trajectories. Together, these results demonstrate that HySimODE produces intuitive species partitions and consistent hybrid trajectories across biologically distinct systems.

Here we introduce **HySimODE**, a machine-learning–guided hybrid simulator that integrates stochastic and deterministic dynamics in a unified Python framework. A deterministic pre-simulation is used to extract dynamical features for each species, after which a pre-trained Random Forest Classifier (RFC) assigns species to stochastic or deterministic regimes. The hybrid engine then integrates both domains concurrently, combining stochastic updates derived from ODE fluxes with stiff ODE solvers within a single event-driven simulation loop (Fig. 1a).

HySimODE operates directly on ODE models rather than explicit reaction networks, and so accepts the same inputs as standard ODE models—equations, parameters, and initial conditions—avoiding any need to reformulate models as reaction networks or define manual thresholds. By automating the partitioning step and reusing ODE formulations directly, HySimODE enables hybrid simulation for multiscale biochemical systems that lie outside the scope of reaction-network–dependent hybrid solvers.

## Methodology

**HySimODE** is a Python framework for hybrid stochastic–deterministic simulation that integrates supervised machine learning with numerical solvers to automate species partitioning in biochemical models. The framework comprises three components: (i) a Random Forest Classifier (RFC) trained on deterministic trajectory simulations, (ii) an application pipeline for unseen models, and (iii) a hybrid integration engine combining stochastic updates derived from ODE fluxes with stiff ODE solvers.

### Random Forest Classifier for species partitioning

The RFC was trained on a curated dataset of 40 biochemical ODE models comprising 294 molecular species spanning deterministic, stochastic-like, and multiscale regimes (including synthetic gene circuits, metabolic systems, and signaling pathways). For each species, deterministic trajectory simulations were used to extract statistical and dynamical features describing amplitude, variability, dynamic range, and temporal behavior (Fig. 1b). Ground-truth labels were assigned using a conservative abundance-based rule: a species was labeled stochastic if the trajectory quantiles satisfied $q_{0.80}(y)<200$ and $q_{0.99}(y)<200$ molecules. Although this rule provides only a coarse approximation, it ensures reproducible labeling and allows the RFC to learn combinations of dynamical features beyond a single cutoff. Classifier performance and generalization were evaluated using leave-one-model-out (LOMO) cross-validation to ensure that predictions were assessed on ODE models not seen during training. Following isotonic probability calibration on the leave-one-model-out (LOMO) fold predictions, probability outputs were well-calibrated and nearly symmetric. Accordingly, a fixed decision threshold of 0.5 was used for all analyses, with no manual tuning. Full details of feature extraction, classifier calibration, hyperparameter search, and training procedure are provided in the Supplementary Information, available as supplementary data at *Bioinformatics* online.

Once trained, the RFC was applied to independent models not included in the training set to evaluate its generalization. Specifically, we tested it on: (i) the *host–repressilator* system (31 ODEs) coupling microbial host physiology to a synthetic genetic oscillator (Weiße et al. 2015); and (ii) a bi-compartmental synaptic tagging and capture (STC) model (23 ODEs) describing long-term potentiation and depression (Smolen et al. 2012). In both cases, the RFC assigned low-copy mRNAs or synaptic tags to the stochastic regime and abundant metabolites or structural proteins to the deterministic regime. These assignments required no manual adjustment. The stochastic/deterministic partition is computed once during initialization and remains fixed throughout the hybrid simulation.

### Hybrid simulator integration

The RFC-guided labels are embedded directly into the **HySimODE** engine. From the user perspective, only an ODE model, initial conditions, and parameters are required. This avoids reformulating models as reaction networks, since the same inputs used for standard deterministic simulations can be used directly. This design allows users to work with standard ODE models without adopting specific reaction-network formalisms (Blinov et al. 2004, Hoops et al. 2006, Lopez et al. 2013, Maarleveld et al. 2013). The workflow proceeds as follows:

1. A deterministic trajectory simulation is run to generate dynamical features for each species.
2. The RFC assigns binary labels (stochastic or deterministic).
3. For each stochastic species, a minimal production–degradation reaction set is automatically derived from its ODE, with propensities evaluated at the current hybrid state. When available, HySimODE can alternatively use an explicit user-provided production–degradation decomposition of the ODE drift to construct stochastic propensities.
4. The hybrid solver alternates between SSA steps for the stochastic subset and stiff ODE integration for the deterministic subset, exchanging state updates within an event-driven loop.

HySimODE therefore operates directly on ODE definitions without requiring users to rewrite models as reaction networks or manually define stochastic–deterministic partitions. The framework supports both *autonomous systems*, where propensities depend only on the molecular state, and *non-autonomous systems*, where explicit time dependence (e.g., pulsed stimuli) is evaluated at the local simulation time. Run-to-run variability can be assessed by repeating simulations. In summary, HySimODE integrates deterministic ODE solvers with stochastic event updates while automating the partitioning step through supervised learning, providing a practical framework for hybrid simulation of multiscale biochemical systems.

### Implementation details

*HySimODE* is implemented in Python using NumPy, SciPy, pandas, and scikit-learn for numerical and machine learning components. The framework includes a modular adapter layer that converts concentration-based ODE models into molecular counts, enabling compatibility with a wide range of biochemical systems. The full implementation, including the pre-trained classifier and example models, is available at https://doi.org/10.5281/zenodo.19503079, with the source code repository hosted at https://github.com/criseidazamora/HySimODE.

## Results

### Automatic partitioning of species by RFC classifier

A central element of HySimODE is its ability to decide which molecular species should be simulated stochastically and which can be treated deterministically. This partition is generated automatically by a Random Forest Classifier (RFC) trained on simulated trajectories. Classifier performance was evaluated using a leave-one-model-out (LOMO) protocol across the curated dataset of 40 biochemical models. Under this evaluation scheme, the classifier achieved high predictive performance, with a mean balanced accuracy of 0.989 and Matthews correlation coefficient (MCC) of 0.982 (Supplementary Table S1, available as supplementary data at *Bioinformatics* online). Performance remained stable across held-out models, indicating that the learned trajectory-derived features generalize to previously unseen biochemical systems. To assess robustness with respect to the abundance threshold used for generating training labels, we performed a threshold-sensitivity analysis in which species were relabeled using thresholds between 150 and 300 molecules. Only a small fraction of species (<2.5%) changed labels relative to the baseline threshold of 200 molecules, and classifier performance remained stable across thresholds (Supplementary Table S5, available as supplementary data at *Bioinformatics* online). Finally, we compared the RFC against simple abundance-based heuristics using trajectory mean or median molecule counts. Across all LOMO folds, the RFC consistently outperformed these baselines, achieving higher balanced accuracy and MCC (Supplementary Table S6, available as supplementary data at *Bioinformatics* online). These results indicate that the classifier captures dynamical signatures of the trajectories beyond simple abundance thresholds.

### The hybrid simulator

The classifier-driven partitioning is embedded within the HySimODE hybrid simulation framework, a Python-based implementation that combines a flux-derived stochastic update rule with stiff ODE solvers. Species labeled as stochastic are simulated using event-driven stochastic updates derived from the local ODE flux, while deterministic species evolve via stiff ODE solvers (LSODA or BDF). HySimODE advances in an event-driven loop alternating SSA firings with deterministic integration, preserving event timing in a piecewise-deterministic Markov process (PDMP) sense. The framework supports both autonomous systems (state-dependent dynamics) and non-autonomous systems with explicit time-dependent inputs. By automating the partition step, HySimODE enables hybrid simulations without manual threshold selection or model reformulation. The stochastic/deterministic assignments are computed once during initialization and remain fixed during the simulation.

### Benchmarking against stochastic and hybrid solvers

To quantitatively evaluate the numerical performance of HySimODE, we compared it against several widely used stochastic and hybrid simulation approaches, including exact SSA, tau-leaping, StochPy, COPASI hybrid simulation, and the Haseltine–Rawlings hybrid method. All methods were applied to the same benchmark system and evaluated using trajectory statistics and runtime measurements (Supplementary Table S9, available as supplementary data at *Bioinformatics* online). As expected, purely deterministic ODE simulations reproduced the mean behavior of the system (e.g., $M_{\text{mean}}=5.000$, $P_{\text{mean}}=2500$) but failed to capture stochastic variability ($M_{\text{var}}=0$, $P_{\text{var}}=0$). In contrast, the reference SSA simulation captured the full stochastic distribution ($M_{\text{var}}=5.02$, $P_{\text{var}}=1.13\times 10^{5}$) with a runtime of approximately 10s. Hybrid approaches reproduced similar statistical properties while reducing computational cost. For example, the Haseltine–Rawlings hybrid solver produced $M_{\text{mean}}=5.009$ and $P_{\text{mean}}=2503.87$ with relative error 0.0147, whereas tau-leaping achieved comparable statistics with relative error 0.0091. HySimODE reproduced both the mean and variance of the stochastic species ($M_{\text{mean}}=5.10$, $M_{\text{var}}=5.26$) while maintaining the correct order of magnitude for the protein statistics ($P_{\text{mean}}=2528.79$, $P_{\text{var}}=1.36\times 10^{5}$), with a relative error of $2.48\times 10^{-2}$. These results demonstrate that HySimODE preserves the stochastic statistics of the reference SSA solution while remaining consistent with existing hybrid solvers. Additional trajectory distributions and runtime analyses are reported in the Supplementary Information (Section 4, available as supplementary data at *Bioinformatics* online).

### Testing on a host–circuit interaction model (31 ODEs)

As a first test case, we applied HySimODE to a *host–repressilator* system coupling microbial resource dynamics with a synthetic genetic oscillator Weiße et al. (2015). The model comprises 31 ODEs describing host and circuit variables. The RFC assigned repressilator mRNAs (mg1, mg2, mg3) to the stochastic regime, while abundant host metabolites and structural proteins were labeled deterministic. Across the 31 species of the model, the classifier identified 22 as stochastic and 9 as deterministic (Supplementary Table S7, available as supplementary data at *Bioinformatics* online). Hybrid simulations exhibited trial-to-trial variability and noisy oscillations in stochastic species, whereas deterministic variables displayed smooth periodic dynamics. HySimODE integrated both regimes concurrently without manual adjustment of the partition (inset in Fig. 1b). This case study illustrates how the hybrid simulator captures intrinsic stochastic fluctuations in low-copy species while maintaining smooth deterministic dynamics for abundant molecular pools (Fig. 1c). Additional hybrid realizations and extended trajectory analyses are provided in the Supplementary Information, available as supplementary data at *Bioinformatics* online.

### Testing on the Smolen synaptic tagging model (23 ODEs)

We next evaluated *HySimODE* on the synaptic tagging and long-term potentiation model proposed by Smolen et al. (2012). The system comprises 23 ODEs describing signaling dynamics across synaptic and dendritic compartments and spans multiple time scales and abundance regimes. HySimODE classified high-abundance species such as Raf, MEK, and ERK as deterministic while assigning low-copy intermediates (e.g., CaMKII and synaptic tags) to the stochastic regime. Across the 23 species of the model, the classifier assigned 16 to the stochastic regime and 7 to the deterministic regime (Supplementary Table S8, available as supplementary data at *Bioinformatics* online). Hybrid simulations reproduced stochastic fluctuations in dendritic species while preserving smooth deterministic dynamics in synaptic variables (Fig. 1d). The concentration-to-molecule adapter handled both compartments using user-defined effective volumes, maintaining correct amplitude scaling for long-term variables such as PRP. The accuracy of the hybrid solver to capture the pulsatile dynamics here depends on the synchronization interval Δt used to couple the deterministic and stochastic components with smaller values of Δt increasing accurately at a computational cost. Further ensemble simulations and species-level trajectory visualizations are reported in the Supplementary Information, available as supplementary data at *Bioinformatics* online.

### Generalization and robustness

In both test cases—neither of which were included in the RFC training set—the classifier produced stochastic–deterministic partitions without manual tuning. This indicates that the trajectory-derived features learned by the RFC generalize to previously unseen biochemical models with comparable dynamical regimes. The resulting partitions were directly used by the hybrid simulator, in which stochastic species were evolved using SSA updates while deterministic species were integrated with stiff ODE solvers within the same event-driven framework. Across both case studies, HySimODE reproduced the expected qualitative behaviors of the underlying deterministic models while introducing stochastic variability in low-copy species. In the host–repressilator system, stochastic fluctuations emerged in mRNA-related species while abundant host variables remained smooth. In the Smolen signaling cascade, stochastic switching events were observed in low-copy intermediates while long-term variables followed the deterministic trajectory. These results demonstrate that HySimODE generalizes across mechanistically distinct biochemical systems—from synthetic gene circuits to intracellular signaling networks—while maintaining consistent hybrid dynamics. Additional trajectory analyses and ensemble simulations are reported in the Supplementary Information, available as supplementary data at *Bioinformatics* online.

## Directions for future development

The performance of HySimODE depends on the ability of the RFC to detect dynamical signatures that distinguish low-copy stochastic species from abundant deterministic ones. Our evaluation indicates that these features generalize across diverse biochemical systems, including metabolic, signaling, regulatory, and oscillatory models. However, the classifier will perform best when new systems exhibit dynamical regimes comparable to those represented in the training dataset. Extremely stiff or ultrasensitive systems may require longer deterministic pre-simulations to ensure that their characteristic dynamics are captured before feature extraction. In the current implementation, species classification is performed once at initialization and remains fixed throughout the hybrid simulation. While this design keeps the framework computationally lightweight, it cannot capture situations where species repeatedly transition between stochastic and deterministic regimes. Supporting dynamic reclassification during simulation would require online feature evaluation and represents an important direction for future development. HySimODE operates directly on the right-hand side of user-defined ODE models, which allows the framework to accommodate systems that cannot easily be expressed as reaction networks. However, this design choice also limits interoperability with SBML-based simulation ecosystems. Providing translation layers or SBML-compatible interfaces therefore represents a natural extension of the current framework. The present implementation assumes Markovian ODE dynamics and does not explicitly incorporate mechanisms with memory, such as transcriptional delays, renewal-type kinetics, or stochastic cell-division events. Extending the classifier and feature set to accommodate these processes represents another promising avenue for future work. Currently these common cell dynamics (e.g. Cao and Grima (2020), Jiang et al. (2021)) could be captured through use of compartments to mimic partitioning at cell division or intermediate states to explicit create delays through direct encoding in the ODEs.

## Conclusions

HySimODE is a hybrid simulation framework for multiscale biochemical models that efficiently integrates multiscale ODE systems composed of stochastic and deterministic variables. The stochastic–deterministic partitioning is automated through a pre-trained Random Forest Classifier (RFC) operating on features extracted from deterministic pre-simulations, thereby eliminating the need for ad hoc abundance thresholds and enabling reproducible, data-driven classification.

We demonstrated the framework on two biologically distinct nonlinear systems: (i) a 31-equation *hostrepressilator* system coupling resource-aware host dynamics with a genetic oscillator, and (ii) a 23-equation bicompartmental synaptic tagging cascade involved in long-term potentiation. These case studies differ markedly in network structure, time scales, and molecular counts, providing representative benchmarks for hybrid simulation. In both systems, HySimODE consistently captured stochastic dynamics in low-copy-number species while preserving the accuracy of the deterministic subsystems.

Beyond these specific benchmarks, HySimODE establishes a systematic and extensible workflow for hybrid simulation. Its minimal requirements—an ODE representation and a short pre-simulation—make it suitable for integration into automated pipelines and high-throughput model screening. The framework operates directly on user-defined ODE models without requiring explicit reaction networks and therefore enables hybrid simulation in settings where propensity-based formulations are unavailable. The modular architecture decouples model specification from simulation logic. Any ODE-based biochemical model can be executed without code changes, enabling hybrid simulation across diverse biological systems.

## Supplementary Material

btag185_Supplementary_Data

## Data Availability

The data and code underlying this article are available in Zenodo at https://doi.org/10.5281/zenodo.19503079. The corresponding source code repository is maintained on GitHub at https://github.com/criseidazamora/HySimODE.
