# Supplementary material for: HySimODE: a hybrid stochastic-deterministic simulation framework for multiscale models of biological systems
Source: Bioinformatics. 2026 Apr 17;42(5):btag185. doi: 10.1093/bioinformatics/btag185 (PMC13141151; doi:10.1093/bioinformatics/btag185)
Supplement: btag185_Supplementary_Data [file btag185_supplementary_data.pdf]

Supplementary Information

HySimODE: A hybrid stochastic-deterministic simulation framework for multiscale models of biological systems

Criseida G. Zamora-Chimal and Alexander P.S. Darlington
School of Engineering, University of Warwick, UK

Correspondence to
criseida.zamora@warwick.ac.uk and a.darlington.1@warwick.ac.uk

This Supplementary Information expands on the methodology presented in the main text. For those only interested in using the framework, we direct them to **Section 6 for examples workflows**.

The Supplementary Information is divided into sections as follows:

- Section 1. Training and evaluation of the Random Forest Classifier (RFC).
- Section 2. Testing of the RFC's prediction capabilities on independent biochemical models.
- Section 3 Integration of the RFC-based classification within the hybrid stochastic-deterministic simulator, including details of the simulator's implementation and the unified model interface which enables model-agnostic execution.
- Section 4. Benchmarking of HySimODE on a classical gene-expression system against established methods.
- Section 5. Demonstration of the RFC-based classification and simulator on unseen models.
- Section 6. User guide and example command-line workflows.
- Section 7. Code and model availability to enable full reproducibility.

Together, this document provides all technical details required to reproduce, extend, and deploy HySimODE across a wide range of user-defined ordinary differential equation models of biological systems.

1 Training of the Random Forest Classifier

To enable automated discrimination between species that should be simulated stochastically and those amenable to deterministic treatment, we trained a Random Forest Classifier (RFC) on a curated dataset of dynamical models. The training procedure consisted of model selection, label definition, feature extraction from simulated trajectories, and classifier calibration.

1.1 Model selection and dataset generation

We constructed a training dataset representative of the molecular-scale regimes relevant to hybrid deterministicstochastic simulation consisting of **40 mechanistic ODE models** spanning **seven functional families**: (1) core metabolism, (2) phosphorylation/signaling cascades, (3) receptorligand systems, (4) deterministic oscillators, (5) gene regulatory circuits, (6) multiscale network motifs, and (7) noise-prone oscillatory systems. All models were processed using a standardized model-audit pipeline (see `audit_models.py` and `audit_family_similarity.py`), which dynamically validates model structure, performs deterministic integration, computes per-species features, assigns regime labels based on full-trajectory abundance statistics, and evaluates feature-space similarity to identify and remove potentially redundant models.

Among the 40 audited models, a total of **294 species** trajectories were analyzed. The model set was intentionally curated such that most models included a mixture of low- and high-copy species, preventing model-level class imbalance and ensuring that the Random Forest Classifier (RFC) encountered diverse dynamical behaviors during training. The dataset of 294 species provides a moderate but informative training set for the Random Forest Classifier, as each species contributes a high signal-to-noise dynamical signature extracted from deterministic simulations. The diversity of kinetic architectures across the 40 curated models compensates for the moderate sample size by exposing the classifier to heterogeneous dynamical regimes. Before final inclusion, redundancy was tested for candidate models using trajectory-derived feature statistics and model-level similarity analysis, ensuring that the benchmark spans diverse dynamical regimes.

For each species, we extracted a compact, reproducible feature set consisting of: (i) whole-trajectory statistics (coefficient of variation, min–max ratio, normalized mean absolute derivative), and (ii) robust window-based descriptors over the initial 50 min and final 100 min segments of each trajectory (medians, coefficients of variation, min–max ratios, and normalized derivatives). Diagnostic quantities (e.g., $q_{0.80}$, $q_{0.99}$ - both defined below - and oscillatory metrics) were retained for auditing but excluded from RFC training.

The resulting 40-model set provides a structured, cross-domain dataset capturing heterogeneity in abundance scales, kinetic architectures, and dynamical behaviors. This diversity is intended to enable the trained RFC to generalize to unseen biochemical systems, including the Smolen model and the hostrepressilator hybrid circuit evaluated in subsequent sections.

1.2 Labeling strategy

Binary labels were assigned using a scale-based criterion derived from abundance quantiles of the deterministic trajectory of each species. A species was labeled as *stochastic* (label 1) if both the 80th and 99th percentiles of its abundance trajectory remained below a threshold of 200 molecules,

$$q_{0.80}(y) < 200 \quad \text{and} \quad q_{0.99}(y) < 200,$$

where $q_p(y)$ denotes the p-quantile of the trajectory $y(t)$ obtained from deterministic ODE simulation. Otherwise, the species was labeled as *deterministic* (label 0).

This rule ensures that species labeled as stochastic remain in the low-copy regime both typically (captured by $q_{0.80}$) and in the high-abundance tail (captured by $q_{0.99}$), preventing trajectories with rare but very large bursts from being classified as deterministic despite exhibiting high-copy excursions.

The resulting dataset is nearly balanced (148 stochastic vs. 146 deterministic species), allowing the Random Forest Classifier to be trained without substantial class imbalance.

1.3 Feature extraction

Feature generation was performed using a standardized pipeline that simulates each model under its deterministic ODE formulation (`make_features_rfc.py`). All trajectories were integrated with the stiff Radau solver (`rtol`=1e–7, `atol`=1e–9) over a fixed horizon of 2000 min to capture both transient and late-time dynamics.

For models defined in concentration units (μM), trajectories were converted to molecule counts prior to feature computation using

$$n = c \times V \times N_A,$$

where c is concentration, V is the compartment volume, and N_A is Avogadro's constant. This ensures consistent physical units across all models and species and allows the extracted features to reflect molecule-scale dynamics relevant for stochastic feasibility.

From each trajectory we extracted a compact set of *scale-aware statistical descriptors*. The features are computed over three temporal regions in order to capture global behaviour, early transients, and late-time dynamics.

Global features. Statistics computed over the full trajectory include the coefficient of variation (`cv_total`), min–max ratio (`minmax_ratio_total`), and normalized mean absolute derivative (`nmadydt_total`). These descriptors summarize overall variability, abundance scale, and characteristic rates of change.

Initial-window features (0–50 min). To characterize early transient behaviour we computed the median abundance (`initial_q50`), coefficient of variation (`initial_cv`), min–max ratio (`initial_minmax_ratio`), and normalized mean absolute derivative (`initial_nmadydt`) within the first 50 minutes of the trajectory.

Final-window features (last 100 min). Analogous descriptors were computed over the final 100 minutes of the simulation: median abundance (`final_q50`), coefficient of variation (`final_cv`), min–max ratio (`final_minmax_ratio`), and normalized mean absolute derivative (`final_nmadydt`). These features capture steady-state levels and residual oscillatory or pulsatile behaviour.

Deterministic feature extraction. All features are extracted from deterministic ODE trajectories rather than stochastic simulations. Deterministic trajectories provide noise-free summaries of the underlying dynamical structure while remaining computationally inexpensive to generate. The stochastic regime information is introduced exclusively through the abundance-based labeling rule (Section 1.2 Labeling strategy), allowing the classifier to learn dynamical signatures associated with molecule-scale regimes without introducing simulation noise into the feature representation.

Simulation horizon. All mechanistic models were integrated deterministically over a standardized simulation horizon of 2000 min. This common horizon was chosen to ensure that transient dynamics, steady states, or multiple oscillatory cycles were fully expressed across the heterogeneous model set. For systems that converge rapidly, trajectories therefore spend a portion of the simulation in steady state, which does not affect the extracted statistics but guarantees consistent temporal coverage across models.

Feature windows are defined relative to this trajectory: an initial window of 0–50 min, full-trajectory descriptors computed over the entire simulation, and final-window descriptors extracted from the last 100 min of the trajectory. This standardized temporal framework ensures that the extracted features capture both early transients and late-time dynamical behaviour in a consistent manner across all models.

Rationale for the 200-molecule threshold Our labelling rule uses a copy-number threshold to distinguish species likely dominated by *intrinsic noise* from those for which a deterministic description is adequate. We set the threshold at 200 molecules based on the following considerations: (i) Noise scaling in the Poisson limit. For birth-and-death-like fluctuations, the intrinsic coefficient of variation (CV) scales as

$$\mathrm{CV}_{\mathrm{intrinsic}} \approx \frac{\sqrt{\mathrm{Var}(N)}}{\mathbb{E}[N]} \approx \frac{1}{\sqrt{N}}. \tag{1}$$

Choosing a reference "small-noise" level of $\mathrm{CV}^\star \approx 0.07$ (i.e., $\sim$7%) gives $N \approx 200$. This is consistent with theoretical and experimental studies on stochasticity in gene expression Gillespie [2000], McAdams and Arkin [1997], Paulsson [2004]. (ii) Validity of diffusion/mean-field approximations. The chemical Langevin equation and mean-field ODE descriptions require

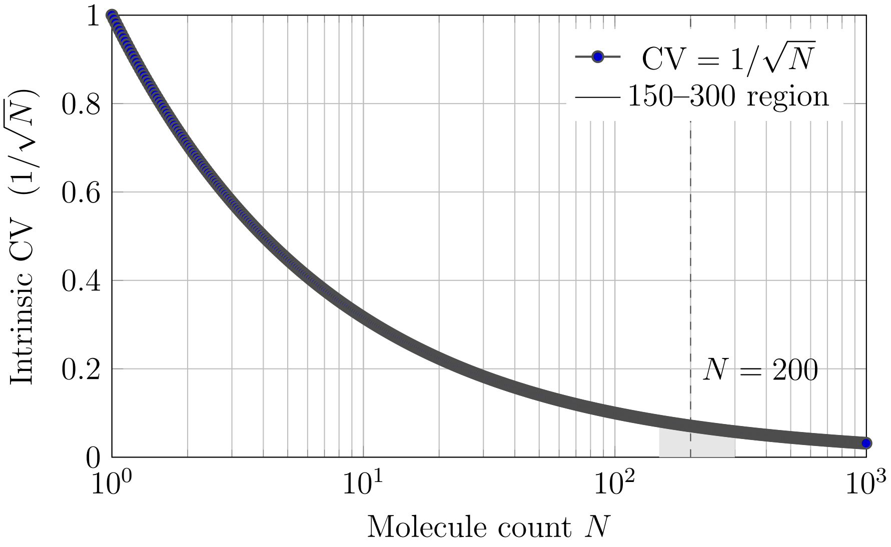

Figure 1: Intrinsic coefficient of variation versus molecule count under Poisson-like fluctuations. The shaded band marks $N \in [150, 300]$; the dashed line highlights the chosen threshold at $N = 200$.

sufficiently large copy numbers and reaction firings per step. Prior work shows that copy numbers in the *hundreds* are typically needed for these approximations to remain accurate Gillespie [2000], van Kampen [2007], Elf and Ehrenberg [2003]. A 200-molecule criterion thus places us on the conservative side of this validity regime. (iii) Empirical robustness on our training set. Thresholds in the range of 150300 molecules yielded nearly identical classifier performance and consistent hybrid partitions. This aligns with experimental and theoretical studies showing that noise is dominant below a few hundred molecules but diminishes at higher abundances Thattai and van Oudenaarden [2001], Pedraza and van Oudenaarden [2005], Shahrezaei and Swain [2008]. We therefore selected 200 as a stable, non-critical value that is both theoretically motivated and empirically supported. (iv) Unit intuition. In a 1 fL compartment, 200 molecules correspond to approximately $0.33\,\mu$M. Many gene-expression species, particularly mRNAs and certain regulatory proteins, naturally reside well below this level, where discreteness and transcriptional bursting make stochastic effects consequential [Taniguchi et al., 2010, Schwanhäusser et al., 2011, Raj and van Oudenaarden, 2008].

1.4 Classifier training

Molecular abundance thresholds alone cannot fully determine whether a species should be simulated deterministically or stochastically, because network structure can strongly modulate noise propagation. For example, ultrasensitivity, positive feedback, and noise amplification mechanisms may produce large fluctuations even at moderate copy numbers, whereas buffering mechanisms can suppress variability in nominally low-copy regimes [Elowitz et al., 2002, Swain et al., 2002, Pedraza and van Oudenaarden, 2005, Paulsson, 2004]. For this reason, the 200-molecule threshold was used only as a heuristic for labeling and consistency checks. The Random Forest Classifier (RFC) then learns nonlinear relationships between

trajectory-derived descriptors (variance, coefficient of variation, slopes, range, and windowed statistics) and the stochasticdeterministic regime, allowing the decision boundary to extend beyond a simple abundance cut-off.

The labeled feature matrix generated above was used to train a Random Forest Classifier (RFC) [Breiman, 2001], implemented in Python 3.12.3 using `scikit-learn` 1.3. All non-numeric identifiers (`model`, `species_name`, `species_index`) were excluded from the feature matrix.

Because multiple species originate from the same mechanistic model and therefore share dynamical and structural dependencies, all validation procedures were carried out at the *model level*, rather than using random splits. This prevents information leakage across folds and ensures a statistically valid evaluation.

Hyperparameters were selected using a *group-aware nested cross-validation* strategy. In the outer loop, we performed **leave-one-model-out (LOMO)** cross-validation, using each mechanistic model as a held-out group. In the inner loop, a randomized hyperparameter search (`n_estimators`, `max_depth`, `min_samples_split`, `min_samples_leaf`, `max_features`, `bootstrap`) was carried out using group-aware cross-validation on the training models only, with macro F1-score as the optimization criterion. This nested procedure yields hyperparameters that generalize across mechanistic models rather than individual observations.

The resulting best-performing group-aware configuration (`n_estimators=600`, `min_samples_-split=4`, `min_samples_leaf=3`, `max_features=log2`, `max_depth=None`, `bootstrap=True`) was retained as the final RFC.

To obtain probabilistic outputs suitable for regime classification, the trained Random Forest was subsequently calibrated using isotonic regression (Section 1.5 Model Calibration). The final calibrated model and associated metadata, including the feature list and selected hyperparameters, were stored as `rfc_calibrated.joblib` and `rfc_metadata.json` to ensure reproducibility.

1.5 Model calibration

Because classifier probabilities are used for stochastic–deterministic partitioning and downstream solver configuration, reliable probability estimates are required. We therefore applied **isotonic regression calibration** using `CalibratedClassifierCV` from `scikit-learn`. Calibration parameters are estimated via cross-validation by fitting a monotonic mapping between the raw classifier probabilities and the observed class labels using isotonic regression.

This approach provides calibrated probability estimates while avoiding overfitting of the calibration model. After calibration, the classifier outputs probabilistic predictions that can be interpreted directly as estimates of the likelihood that a species should be treated as stochastic.

Given this probabilistic interpretation, the final classifier uses a fixed classification threshold of **0.5**. This threshold provides a transparent and reproducible decision rule across

heterogeneous mechanistic models and avoids manual tuning of classification cutoffs.

The calibrated classifier, together with the selected hyperparameters, feature definitions, and fold-level evaluation results, is stored as `rfc_calibrated.joblib` and `rfc_metadata.json` to ensurefull reproducibility.

2 Testing of the Random Forest Classifier

2.1 Evaluation metrics of RFC

Performance was assessed using a comprehensive set of metrics that capture discrimination, calibration, and class-specific behavior across heterogeneous biochemical models. Because species originating from the same mechanistic model are not statistically independent, all reported metrics were computed in a *model-grouped* manner using the outer leave-one-model-out (LOMO) folds.

For each held-out model, we calculated: (i) **balanced accuracy**, which provides an unbiased estimate under class imbalance by averaging the true positive rates of the deterministic and stochastic classes; (ii) the **Matthews correlation coefficient** (MCC), a robust summary of binary classification quality even for extreme imbalance; (iii) **ROC–AUC** and **average precision**, computed only when both classes were present in the held-out fold; and (iv) class-wise **precision**, **recall**, and **F1-score**. Macro-averaged F1 was also computed to provide a class-independent summary.

To avoid misleading metrics in monoclass folds, the evaluation procedure follows the conventions of `scikit-learn`: ROC–AUC, average precision, balanced accuracy, and MCC are set to `NaN` when the held-out fold contains only one class. These values are excluded from summary statistics but preserved in the per-model results for transparency. All metrics were aggregated over the 40 LOMO folds, reporting mean and standard deviation.

2.2 Robustness analysis and freezing of the classifier

To ensure that the trained classifier could be reliably deployed within the hybrid simulation framework, we performed a series of robustness analyses under the leave-one-model-out (LOMO) evaluation protocol.

LOMO generalization performance Classifier performance was first assessed using leave-one-model-out (LOMO) cross-validation across 40 mechanistic biochemical models. In this evaluation scheme, all species belonging to a given model were held out simultaneously, ensuring that the classifier was tested only on models not seen during training.

Classifier performance under the LOMO protocol is summarized in Table 1. Under this evaluation scheme, the classifier achieved high predictive performance, with a mean balanced accuracy of 0.989, Matthews correlation coefficient (MCC) of 0.982, and an F1-score of 0.918 for the stochastic class. ROC–AUC and average precision were equal to 1.0 in all folds

where both classes were present. These results indicate that the classifier generalizes well to previously unseen biochemical models.

Some biochemical models contain only one class of species, making threshold-free metrics such as ROC–AUC and MCC undefined for those folds. The coverage of valid metrics across LOMO folds is summarized in Table 2.

Nested LOMO validation To assess potential bias introduced by global hyperparameter selection, we performed a nested LOMO evaluation in which hyperparameter optimization was repeated independently within each outer fold using group-aware cross-validation on the training models only. The resulting performance metrics were indistinguishable from those obtained using the globally selected hyperparameters, indicating that classifier performance is stable with respect to the hyperparameter tuning procedure and that the reported LOMO results are not inflated by selection bias.

Bootstrap confidence intervals Statistical uncertainty in the estimated performance was quantified using bootstrap resampling over held-out models, treating each biochemical model as the independent statistical unit. Across 2000 bootstrap resamples, the 95% confidence interval for the MCC ranged from 0.95 to 1.00, while the balanced accuracy remained above 0.97. These results confirm that the classifier maintains consistently high predictive accuracy across diverse model structures.

Permutation tests To verify that classifier performance could not be explained by chance correlations in the feature set, we conducted permutation tests under the LOMO protocol using 500 permutations. Two permutation schemes were considered: global permutation of labels across the dataset and permutation within each biochemical model, which preserves the class structure of each model.

The resulting null distributions and statistical significance values are reported in Table 3. Under label permutation, the null models produced mean MCC values close to zero, whereas the observed classifier achieved MCC = 0.982. The difference between observed and permuted performance was statistically significant ($p < 0.002$). Similar results were obtained under both permutation schemes, demonstrating that the classifier captures genuine signal rather than spurious correlations.

Freezing of the classifier Taken together, these analyses demonstrate that the classifier exhibits strong and stable predictive performance across heterogeneous biochemical models, with consistent results across nested cross-validation, bootstrap resampling, and permutation testing. Based on this evidence, the final calibrated random forest classifier was trained on the full dataset using the selected hyperparameters and subsequently frozen for integration into the hybrid simulation framework.

Family-level generalization (LOFO) As an additional robustness analysis, we evaluated the classifier using a leave-one-family-out (LOFO) protocol in which entire families of

biochemical models (metabolic, signalling, gene regulatory, oscillatory, receptorligand, and related systems) were held out during training.

Under this evaluation scheme, the classifier achieved a mean balanced accuracy of 0.970 and MCC of 0.918 across seven model families (Table 4), with ROC–AUC = 0.993 and average precision = 0.987. Although performance is slightly lower than in the LOMO evaluation, this protocol represents a more challenging setting in which the classifier must generalize to classes of biochemical systems not observed during training.

These results indicate that the learned trajectory-derived features capture dynamical signatures that remain consistent across diverse biochemical network architectures.

Table 1: Classifier performance under the leave-one-model-out (LOMO) protocol. Mean and standard deviation are computed across held-out models. Bootstrap confidence intervals were estimated using 2000 resamples treating each model as an independent unit.

Metric	Mean	Std	95% CI (bootstrap)
Accuracy	0.986	0.054	[0.966, 1.000]
Balanced accuracy	0.989	0.047	[0.970, 1.000]
MCC	0.982	0.079	[0.950, 1.000]
Macro F1	0.977	0.098	[0.941, 1.000]
Precision (stochastic)	0.914	0.269	[0.819, 0.989]
Recall (stochastic)	0.925	0.267	[0.825, 1.000]
F1 (stochastic)	0.918	0.267	[0.823, 0.993]
ROC–AUC	1.000	0.000	[1.000, 1.000]
Average precision	1.000	$< 10^{-16}$	[1.000, 1.000]

Table 2: Coverage of evaluation metrics across LOMO folds. Some models contain only one class (deterministic or stochastic), in which case threshold-free metrics such as ROC–AUC and MCC are undefined and reported as NaN.

Statistic	Value
Total models	40
Binary folds	34
Monoclass folds	6
Folds with defined MCC	34
Folds with defined ROC–AUC	34
Folds with defined average precision	34

2.3 RFC testing via a threshold sensitivity analysis

To evaluate the robustness of the Random Forest Classifier (RFC) with respect to the abundance threshold used for generating training labels, we performed a dedicated threshold-sensitivity analysis based on the deterministic feature matrix (`features_rfc.csv`). Species

Table 3: Permutation test results under the LOMO protocol using 500 permutations. Two null models were considered: global permutation of labels and permutation within each biochemical model.

Metric	Observed	Null mean (global)	Null mean (within-model)	p-value
Accuracy	0.986	0.497	0.641	0.002
F1 (stochastic)	0.918	0.447	0.497	0.002
MCC	0.982	-0.003	-0.003	0.002

Table 4: Leave-one-family-out (LOFO) evaluation across biochemical model families. Each fold trains the classifier on all families except one and tests on the held-out family.

Metric	Mean ± SD
Accuracy	0.963 ± 0.038
Balanced accuracy	0.970 ± 0.031
Macro F1	0.956 ± 0.041
MCC	0.918 ± 0.074
ROC–AUC	0.993 ± 0.009
Average precision	0.987 ± 0.020
F1 (stochastic)	0.960 ± 0.038

labels were re-assigned using the same quantile-based rule employed in the main pipeline,

$$\text{label} = \begin{cases} 1, & \text{if } q_{80} < T \text{ and } q_{99} < T, \\ 0, & \text{otherwise,} \end{cases} \tag{2}$$

where q_{80} and q_{99} denote the 80th and 99th percentiles of the species-level molecule-count trajectory. We tested

$$T \in \{150,\ 200,\ 250,\ 300\} \text{ molecules,}$$

covering a conservative range around the default threshold of $T = 200$ used in the main text.

For each value of T, the classifier was evaluated using *leave-one-model-out* (LOMO) cross-validation, treating each mechanistic model as an independent group and holding out all species from one model per fold. The RFC architecture and hyperparameters were kept fixed to those selected by the group-aware search in the main analysis:

$$\begin{aligned} \texttt{n\_estimators} &= 600, \\ \texttt{min\_samples\_split} &= 4, \\ \texttt{min\_samples\_leaf} &= 3, \\ \texttt{max\_features} &= \text{"log2"}, \\ \texttt{max\_depth} &= \text{None}, \\ \texttt{bootstrap} &= \text{True}. \end{aligned}$$

Table 5 summarizes, for each T: (i) the number and percentage of species whose labels differ from the baseline $T = 200$ assignment, and (ii) the mean and standard deviation of the balanced accuracy and Matthews correlation coefficient (MCC) across the 40 LOMO folds.

Only 0.68%-2.38% of species change labels relative to $T = 200$, and the classifiers performance remains highly stable across thresholds (balanced accuracy: 0.970 ± 0.066 to 0.989 ± 0.047; MCC: 0.942 ± 0.123 to 0.982 ± 0.079). These results demonstrate that the RFC is not relying on a sharp abundance cut-off but instead is exploiting more robust dynamical signatures derived from the deterministic pre-simulation.

A reproducible implementation of this analysis is provided in the script `threshold_sensitivity_rfc.py` (available in the project repository), which regenerates all results in Table 5.

Table 5: Sensitivity of RFC performance to the abundance threshold T. For each T, species were relabeled using the rule in Eq. 2 and evaluated with LOMO cross-validation.

T (mol.)	Label flips	% flips	Balanced accuracy (mean ± SD)	MCC (mean ± SD)
150	7 / 294	2.38%	0.970 ± 0.066	0.942 ± 0.123
200	0 / 294	0.00%	0.989 ± 0.047	0.982 ± 0.079
250	2 / 294	0.68%	0.978 ± 0.058	0.953 ± 0.122
300	4 / 294	1.36%	0.980 ± 0.057	0.962 ± 0.111

2.4 Comparison with simple abundance heuristics

To evaluate whether the Random Forest Classifier (RFC) provides predictive information beyond simple abundance thresholding, we compared its performance against two commonly used heuristic rules based solely on molecule abundance statistics. These heuristics classify a species as stochastic if its abundance is below a fixed threshold of 200 molecules:

$$\text{mean heuristic: } \text{mean}(y) < 200, \qquad \text{median heuristic: } \text{median}(y) < 200,$$

where mean(y) and median(y) denote the mean and median molecule counts computed over the deterministic trajectory of each species.

Ground-truth labels were defined using the quantile-based rule described in Section 1.2 Label strategy (based on the trajectory quantiles q_{80} and q_{99} with threshold $T = 200$). Importantly, these quantities were not used as input features for the RFC, preventing trivial reconstruction of the labeling rule.

For a fair comparison, heuristic predictions were evaluated under the same leave-one-model-out (LOMO) cross-validation protocol used for the RFC and on the same curated dataset of 40 mechanistic models spanning diverse biochemical network families. Specifically, at each fold a single mechanistic model was held out, and predictions were generated for all species belonging to that model. The RFC was trained on the remaining models, whereas heuristic predictions were obtained directly from the abundance rule without training. Performance metrics (balanced accuracy and Matthews correlation coefficient, MCC) were then computed on the held-out model.

This evaluation framework ensures that the RFC and heuristic classifiers are assessed on identical held-out species and that statistical dependence between species originating from the same mechanistic model is respected.

Across the full dataset, the RFC consistently outperformed both heuristic baselines based on mean and median molecule-count thresholds (Table 6). These results indicate that the classifier captures dynamical features of the trajectories that cannot be explained by simple abundance thresholds alone.

Table 6: RFC performance compared with simple abundance-threshold heuristics under the leave-one-model-out (LOMO) protocol. Heuristic rules classify a species as stochastic if either the trajectory mean or median molecule count falls below a threshold T. Metrics are reported as mean ± SD across LOMO folds.

Method	Balanced accuracy	MCC
RFC (calibrated; $p \geq 0.5$)	$\mathbf{0.983 \pm 0.052}$	$\mathbf{0.963 \pm 0.107}$
Mean < 150	0.954 ± 0.082	0.920 ± 0.131
Mean < 200	0.923 ± 0.125	0.861 ± 0.223
Mean < 250	0.923 ± 0.125	0.861 ± 0.223
Median < 150	0.887 ± 0.125	0.803 ± 0.219
Median < 200	0.878 ± 0.138	0.781 ± 0.252
Median < 250	0.874 ± 0.137	0.770 ± 0.251

2.5 RFC testing via external validation on unseen biochemical models

To assess cross-domain generalization, we evaluated the calibrated RFC on two multi-scale biochemical systems that were not used during training, hyperparameter selection, probability calibration, or threshold determination: (i) a host–repressilator interaction model, and (ii) the Smolen synaptic tagging model.

This constitutes a strict external test. However, because these systems lack ground-truth stochastic/deterministic assignments, our goal is not estimation accuracy but rather a quantitative characterization of classifier behaviour on out-of-distribution inputs. All predictions were made using the calibrated RFC with a fixed decision threshold of 0.5.

The host–repressilator model comprises **31** species. The classifier assigned **22** species (**71.0%** of species) to the stochastic regime and **9** species (**29.0%**) to the deterministic regime. Detailed quantitative statistics, including mean and median calibrated probabilities and the 10–90% probability range, are provided in **Table 7**. Calibrated probabilities exhibit a strongly bimodal structure (mean ± SD: **0.698 ± 0.454**; median: **0.964**), indicating high classifier confidence consistent with the systems known multiscale architecture.

The Smolen model includes **23** species, of which the classifier assigned **16** (**69.6%** of species) to the stochastic regime and **7** (**30.4%**) to the deterministic regime. Quantitative statis-

tics for this system are reported in **Table 8**. In contrast to the host–repressilator system, calibrated probabilities are more broadly distributed (mean $\pm$ SD: **0.641** $\pm$ **0.456**; median: **0.816**), reflecting the coexistence of stable high-copy components and transient, noise-amplifying signaling intermediates.

Together, these results demonstrate that the classifier behaves consistently across mechanistically diverse systems: it produces high-confidence, bimodal assignments in models with clear scale separation, and more graded probability profiles in systems with mixed-regime signaling dynamics. This supports the interpretation that the RFC captures transferable dynamical signatures rather than model-specific artifacts.

Table 7: Quantitative classifier statistics for the host–repressilator external evaluation model. All predictions made using the calibrated RFC with threshold 0.5.

**Metric**	**Value**
Total species	31
Stochastic (label = 1)	22
Deterministic (label = 0)	9
Mean P(stoch)	0.698
Median P(stoch)	0.964
10–90% range	0.0 – 1.0
Minimum P(stoch)	0.0
Maximum P(stoch)	1.0

Table 8: Quantitative classifier statistics for the Smolen synaptic tagging external evaluation model. All predictions made using the calibrated RFC with threshold 0.5.

**Metric**	**Value**
Total species	23
Stochastic (label = 1)	16
Deterministic (label = 0)	7
Mean P(stoch)	0.641
Median P(stoch)	0.816
10–90% range	0.0 – 1.0
Minimum P(stoch)	0.0
Maximum P(stoch)	1.0

3 Outline of the Hybrid Simulator

We incorporated the RFC predictions into a hybrid simulation framework that couples deterministic ordinary differential equation (ODE) solvers with stochastic simulation algorithms (SSA). This integration enables species-level resolution, where some variables are simulated deterministically while others follow exact stochastic dynamics within the same system.

Importantly, HySimODE does *not* reconstruct or assume an underlying chemical reaction network. All computations operate directly at the level of species-wise ODE drifts. The method never infers reaction channels, stoichiometric matrices, nor mass-balanced chemistry underlying the model. This distinction is critical to avoid misinterpretation of the productiondegradation decomposition described below.

3.1 Hybrid simulation framework

HySimODE is integrated by two stages:

Stage 1. RFC Classification The feature extraction needed for the RFC model relies on a deterministic pre-simulation of the ODE system performed over the same time horizon as the hybrid simulation ($0 \leq t \leq T_{\text{final}}$). The resulting trajectories are used to compute the time-series features required by the Random Forest classifier. Species are then assigned to deterministic or stochastic regimes prior to the hybrid simulation. In the current implementation, this assignment remains fixed during the simulation. While dynamic re-evaluation of regimes could be considered in future extensions, the static classification was found to be sufficient for the systems analysed in this study.

Stage 2. Hybrid Simulation. After the species classifications is performed by the RFC classifier the hybrid simulations comes up. Time simulation is partitioned into fixed blocks of duration Δt. Within each block, HySimODE performs two complementary updates:

1. **Stochastic SSA step (species-wise birth–death process).**

   For each species classified as stochastic, the model provides a decomposition of its ODE drift f_i into non-negative production and degradation fluxes,

$$f_i(x,t) \;=\; \text{prod}_i(x,t) \;-\; \text{deg}_i(x,t), \qquad \text{prod}_i, \text{deg}_i \geq 0.$$

   These quantities should *not* be interpreted as reaction propensities associated with underlying biochemical reactions. Instead, they are algebraic, species-level fluxes used solely to define a two-channel birthdeath process for each stochastic species. This construction guarantees that the expected drift of the stochastic update matches the original ODE exactly.

   The SSA step therefore uses only the aggregated intensities

$$a_i^{\text{prod}} = \text{prod}_i, \qquad a_i^{\text{deg}} = \text{deg}_i,$$

which generate ±1 changes in the species count. HySimODE does not attempt to infer a reaction network and no additional reaction channels, stoichiometric couplings, or conservation-law constraints are introduced. Concerns such as breaking mass balance or introducing independent Poisson processes for hypothetical conversions do not arise, as the stochastic mechanism operates directly at the species level and is constructed to preserve the deterministic drift component-wise.

2. **Deterministic ODE step for the remaining species.**

   After the SSA updates, the deterministic subset is integrated over $[t,\, t + \Delta t]$ using the reduced ODE system in which stochastic coordinates are held fixed. We employ `solve_ivp` with a stiff implicit method (typically `Radau` or `BDF`), using `rtol` $= 10^{-6}$, `atol` $= 10^{-9}$, and `max_step` $\leq \Delta t$ to ensure numerical stability in systems with disparate time scales and to avoid overshooting stochastic events.

This blockwise coupling preserves the exact ODE drift for all species, introduces biophysically meaningful noise only on the RFC-selected subset, and maintains synchronization between stochastic and deterministic components throughout the simulation.

3.2 Net drift and production–degradation decompositions

HySimODE is designed to operate directly from an ODE model, whose primary input is the species-wise drift $f_i(x,t)$. By default, the hybrid simulator constructs stochastic propensities using only this net drift, since any species-level birth–death process satisfying

$$\mathbb{E}[\Delta x_i]/\Delta t = f_i$$

preserves the mean behaviour of the ODE exactly. This "netdrift mode" provides a minimal and fully general stochastic representation for arbitrary deterministic models, requiring no additional information from the user.

When available, HySimODE can also incorporate an explicit production–degradation decomposition supplied by the user,

$$f_i(x,t) \;=\; \mathrm{prod}_i(x,t) \;-\; \mathrm{deg}_i(x,t), \qquad \mathrm{prod}_i, \mathrm{deg}_i \geq 0.$$

In this case, the simulator uses

$$a_i^{\mathrm{prod}} = \mathrm{prod}_i(x,t), \qquad a_i^{\mathrm{deg}} = \mathrm{deg}_i(x,t),$$

which reflect the underlying turnover implied by the models ODE formulation. Both approaches reproduce the deterministic drift exactly, but they may differ in the resulting noise amplitude: in some systems, using the explicit production and degradation fluxes generates higher variability, whereas in others both approaches yield comparable fluctuation levels. HySimODE therefore supports both modes so that users can select the level of stochastic detail appropriate for their application.

3.2.1 User-defined interfaces for stochastic propensity evaluation

In the hybrid simulation framework, stochastic propensities are computed by the function `compute_propensities()`. This function translates the deterministic description of the system into nonnegative propensities required by the stochastic simulation algorithm (SSA). To support models of different levels of mechanistic detail, the framework allows two alternative user-defined interfaces for specifying reaction rates.

Minimal interface: net deterministic dynamics. The minimal requirement for any model used in the framework is the definition of the ordinary differential equation (ODE) system

`odes(t, y, params)`

which returns the time derivative of the state vector y. When only this function is provided, the framework approximates stochastic propensities directly from the net deterministic drift of each species.

For a species i with deterministic derivative

$$\frac{dy_i}{dt} = f_i(t, y),$$

the effective production and degradation propensities are constructed by separating the positive and negative components of the drift:

$$a_i^{\text{prod}} = \max\big(f_i(t, y),\, 0\big),$$

$$a_i^{\text{deg}} = \max\big(-f_i(t, y),\, 0\big).$$

The positive component of the drift is interpreted as an effective production rate, while the negative component corresponds to an effective degradation rate. This approximation enables hybrid simulations even when the user only provides the deterministic model without an explicit reaction decomposition.

Optional interface: explicit production–degradation decomposition. For models where a clearer mechanistic interpretation is desired, the user may optionally define an additional function

`odes_prod_deg(t, y, params)`

which returns two vectors

$$\mathbf{p}(t, y), \quad \mathbf{d}(t, y),$$

representing the total production and degradation contributions for each species. These vectors must satisfy

$$p_i \geq 0, \qquad d_i \geq 0,$$

and have the same dimensionality as the state vector.

When this function is available, it is used preferentially to construct the stochastic propensities:

$$a_i^{\text{prod}} = p_i(t, y), \qquad a_i^{\text{deg}} = d_i(t, y).$$

This formulation allows the stochastic simulator to operate on rates that directly correspond to the mechanistic structure of the biochemical model.

The framework additionally performs basic consistency checks, including dimensional verification and optional validation that the deterministic drift satisfies approximately

$$f_i(t, y) \approx p_i(t, y) - d_i(t, y).$$

Providing `odes_prod_deg()` is therefore recommended when the production and degradation processes of the model can be explicitly separated, although it is not strictly required for running hybrid simulations.

Crucially, in either mode, HySimODE does not reconstruct a chemical reaction network or impose reaction-level constraints. All updates operate directly at the species level and preserve the drift of the original ODE system component-wise.

3.3 Special considerations

Models defined in concentration units. Some biochemical models are formulated in concentration units (μM) rather than molecule numbers. Since stochastic simulation algorithms and the RFC classifier operate on discrete molecular populations, HySimODE includes a generic adapter (`concentration_adapter_hybrid.py`) that converts concentration-based ODE models into molecule counts prior to hybrid simulation. The adapter performs the conversion using compartment volumes defined by the model and allows existing concentration-based ODE models to be used directly without modifying the original model equations.

For multi-compartment systems, the model can optionally specify compartment structure using the generic interfaces `compartment_indices` (mapping species to compartments) and `compartment_volumes_L` (defining compartment volumes in liters). This information enables the adapter to compute the correct molecule scaling for each species before stochastic simulation.

Autonomous and non-autonomous models. Classical stochastic simulation algorithms (SSA), including Gillespies Direct Method, are typically formulated for autonomous systems in which reaction propensities depend only on the molecular state vector. However, many biochemical models include explicit time-dependent inputs such as external stimuli or pulsed signals. In such cases the system becomes non-autonomous, and propensities depend on both the state and time [Anderson, 2007].

In HySimODE, stochastic propensities are derived from species-level production and degradation intensities obtained from the ODE system. When the ODE contains explicit time-dependent inputs (e.g., pulsed Ca^{2+} elevations in the Smolen synaptic-tagging model), these intensities must be evaluated at the current stochastic time t_{local}. HySimODE therefore evaluates propensities as $a_i(t_{\text{local}}, y)$, where t_{local} denotes the current stochastic simulation time. This allows the stochastic component to remain consistent with the underlying ODE model when explicit time-dependent inputs are present.

3.4 Pseudocode

The pseudocode below summarizes the HySimODE workflow, including RFC-based partitioning, SSA/ODE coupling, and the species-wise production/degradation construction of propensities.

```
# --- Model and classifier setup ---
load model: odes(t, y, params), Y0, params, var_names
load optional solver_options, otherwise use defaults
load calibrated RFC: (clf, feature_names, prob_threshold)

# --- Deterministic pre-simulation and species classification ---
t_eval = linspace(0, T_FINAL, floor(T_FINAL/DT)+1)
sol_det = solve_ivp(lambda t, y: odes(t, y, params),
                    [0, T_FINAL], Y0, t_eval=t_eval, **solver_options)
Y_det = maximum(sol_det.y, 0.0)

stoch_idx, decisions = classify_species_with_rfc(
    sol_det.t, Y_det, clf, feature_names, prob_threshold=prob_threshold
)

# --- Build species-wise birth-death layer for stochastic species ---
for each i in stoch_idx:
    add reaction (i, 'prod') with +1 stoichiometric change
    add reaction (i, 'deg')  with -1 stoichiometric change

# --- Reduced deterministic subsystem ---
def reduced_system(t, y):
    dy = odes(t, y, params)
    set dy[i] = 0 for all i in stoch_idx
    return dy

# --- Hybrid simulation loop ---
for run in range(NUM_RUNS):
    t = 0
    state = copy(Y0)
```

```
store initial state

while t < T_FINAL:
    t_end = min(t + DT, T_FINAL)
    t_local = t

    # SSA updates within current block
    while True:
        compute propensities:
            if odes_prod_deg is available:
                use explicit production/degradation decomposition
            else:
                use positive/negative parts of the net ODE drift

        a0 = sum(propensities)
        if a0 <= 0:
            break

        tau = exponential(1/a0)
        if t_local + tau > t_end:
            break

        select reaction according to normalized propensities
        update state with the selected +/-1 jump
        enforce state >= 0
        t_local += tau

    # ODE update on deterministic species
    sol_block = solve_ivp(reduced_system, [t, t_end], state, **
       solver_options)
    state = sol_block.y[:, -1]
    t = t_end
    store block-end state

save run outputs
```

Listing 1: Pseudocode of the learning-based hybrid ODE/SSA simulation framework

3.5 HySimODE simulation outputs

Each simulation run produces full time series for all species, combining deterministic and stochastic trajectories. All results are saved under `output_hybrid/`, including subdirectories for replicate runs (`runs/`), numerical trajectories (`timeseries_txt/`), and graphical plots. The RFC output file (`rfc_decisions.csv`) stores per-species probabilities and labels. Post-processing with `analyze_runs.py` aggregates replicate trajectories to compute mean and standard deviation profiles over time and can optionally generate ensemble statistics and plots for publication.

3.6 Complete unified model interface and execution workflow

HySimODE provides a unified execution interface that enables any biological model formulated as an ODE system to be simulated in hybrid stochastic–deterministic mode without model-specific modifications. The workflow abstracts three core components: a base model, a unit-conversion adapter, and an execution engine. This modular design ensures that both concentration-based and molecule-based models can be executed seamlessly within the same framework.

The execution engine (`hysimode.py`) dynamically loads user-specified models using Python's `importlib` mechanism, requiring only that each model file defines an ODE function (`odes`), initial conditions (`Y0`), parameters (`params`), and variable names (`var_names`). Once loaded, the engine performs a deterministic pre-simulation using `solve_ivp` (method = `BDF`, `rtol` = 10^{-6}, `atol` = 10^{-9}, `max_step` = 0.02) to generate baseline trajectories. These are used to compute per-species features and the calibrated Random Forest Classifier (RFC) is applied through `rfc_integration.py` to produce a CSV file with probabilities and binary regime labels (`output_hybrid/rfc_decisions.csv`). When available, an optional user-defined function `odes_prod_deg` can be provided to supply an explicit production–degradation decomposition of the ODE fluxes. These quantities are then used for stochastic propensity evaluation, as described above in Section 3.2 and illustrated in the example `hysimode/models/gene_expression_odes_prod_deg.py`, which is also used as the benchmark model in Table 9.

Species classified as stochastic are assigned minimal birthdeath reactions as described above, while deterministic species are integrated with a reduced ODE system where their stochastic counterparts are frozen. The resulting hybrid simulation alternates SSA and ODE blocks over fixed intervals (Δt), preserving consistency between stochastic and deterministic subsystems. For concentration-based models, the adapter `concentration_adapter_hybrid.py` automatically rescales concentrations to molecule counts prior to simulation, requiring no manual code edits.

Simulations are launched from the command line with a single invocation, e.g.:

```
python hysimode.py --model host_repressilator.py --tfinal 2000 --dt 1 --runs 20
```

or, for concentration-based models, e.g.:

```
BASE_MODEL=smolen_odes \
python hysimode.py --model concentration_adapter_hybrid.py \
                   --tfinal 500 --dt 0.01 --runs 20
```

Each execution produces structured outputs under `output_hybrid/`, including time-series data (`timeseries_txt/`), per-run results (`runs/`), and trajectory plots for all species. Post-simulation analysis is performed by `analyze_runs.py`, which aggregates replicate trajectories to compute per-time means and standard deviations and can generate ensemble statistics and figures. Together, these components establish a reproducible, fully automated workflow linking model inference, hybrid regime prediction, and simulation within a single command-line interface. Overall, HySimODE provides a generalizable bridge between mechanistic modeling and data-driven hybrid simulation. By combining machine learning-based regime

classification with automated solver orchestration, it enables reproducible, scalable, and adaptive simulation of biochemical systems under mixed stochastic-deterministic regimes.

4 Benchmarking of HySimODE on a classical gene-expression system

To establish a quantitative performance baseline applicable across ODE-defined biochemical systems, we benchmarked HySimODE against deterministic ODE simulation and exact stochastic simulation (SSA) using a standard two-stage gene expression model,

$$\frac{dM}{dt} = \alpha - \delta_m M, \qquad \frac{dP}{dt} = \beta M - \delta_p P.$$

This model admits both a four-reaction SSA formulation and a closed-form analytic ODE solution, providing a controlled setting in which deterministic, stochastic, and hybrid simulation schemes can be compared directly without requiring network reconstruction. All benchmarks were produced using independent scripts for each method. For every simulator, we generated 200 independent trajectories and evaluated final-state distributions, trajectory means and variances, and wall-clock runtime, enabling a balanced assessment of the accuracy and computational performance of HySimODE relative to ODE and SSA.

The implementation of this benchmark model used in the HySimODE simulations is provided in `hysimode/models/gene_expression.py` in the public repository.

We employed the following methods in benchmarking HySimODE:

- **ODE**: Analytical deterministic trajectories computed on a fixed time grid, representing the mean-field limit of the system.
- **SSA**: Gillespie direct method using the canonical reaction decomposition of the gene-expression model, providing the exact stochastic reference distribution Gillespie [1977].
- **StochPy**: External implementation of the Gillespie SSA algorithm, executed via the StochPy 2.5.0 Python interface Maarleveld et al. [2013] to provide an independent stochastic baseline.
- **COPASI Hybrid**: Hybrid stochastic–deterministic simulation performed with the COPASI time-course task Hoops et al. [2006]. Fast reactions are integrated deterministically using LSODA, while low-copy-number species follow an SSA-based jump process. This method serves as a well-established hybrid baseline for comparison against HySimODE.
- **Tau_leap**: Adaptive tau-leaping scheme following the approach of Cao, Gillespie, and Petzold, using Poisson-distributed reaction firings and automatic step-size control Cao et al. [2005].
- **Hybrid_HR**: A classical Haseltine–Rawlings hybrid method in which mRNA dynamics are simulated stochastically (SSA) while protein dynamics evolve deterministically between events (piecewise-analytic solution) Haseltine and Rawlings [2002].
- **HySimODE-netdrift**: Full hybrid simulation using a Random Forest Classifier (RFC) for adaptive, state-dependent species partitioning. Species classified as stochastic are

simulated via SSA, while deterministic species evolve via ODE integration. Propensities are computed from the *net drift* of ODE fluxes, aggregating all production and degradation contributions.

- **HySimODE**: Full hybrid simulation using RFC-based, state-dependent species partitioning. As in HySimODE-netdrift, SSA is applied to stochastic species and ODE integration to deterministic ones. Here, propensities are derived from *signed* ODE flux components, separating positive (production) and negative (consumption) contributions to achieve a more resolved hybridization.

4.1 Final distribution statistics

Table 9 summarises the mean and variance of M and P at the final simulation time for all benchmarked methods. SSA provides the reference values for both statistics. StochPy and tau-leaping achieve close agreement with SSA in mean and variance, with tau-leaping offering a substantially faster runtime. COPASI Hybrid reproduces the SSA mean behaviour but slightly underestimates the variance of both species, consistent with the deterministic treatment of selected reaction channels. Deterministic approaches (ODE) produce zero variance because stochastic fluctuations are not explicitly simulated.

HySimODE-netdrift matches the deterministic mean values exactly, as expected from its use of net drift for propensity reconstruction, but suppresses stochastic variability. In contrast, HySimODE captures both the stochastic spread and the central moments with high fidelity, achieving accuracy comparable to StochPy while maintaining a hybrid formulation. Runtime measurements further show that HySimODE provides a favourable trade-off between accuracy and computational cost compared with fully stochastic approaches.

Table 9: Final-time distribution statistics for the gene-expression benchmark. Means and variances were computed over 200 independent trajectories. Relative errors for protein variance are reported with respect to the SSA reference. Runtime corresponds to total wall-clock time, in seconds, for generating 200 trajectories for each method.

**Method**	M **mean**	M **var**	P **mean**	P **var**	**Rel. err.**	**Runtime**
ODE	5.0000	0.0000	2500.00	0.0000	0.0131156	6.97E-05
SSA	5.0205	5.0216	2467.64	113068.92	0	9.98
StochPy	4.9611	4.9346	2488.07	124146.58	0.0082823	194.25
Tau_leap	4.9450	5.2805	2489.99	114135.77	0.0090618	4.52
Copasi Hybrid	4.9792	4.7394	2514.56	114305.80	0.0190186	0.04
Hybrid_HR	5.0090	4.9125	2503.87	106185.80	0.0146849	0.2030
HySimODE-netdrif	5.0000	0.0000	2500.00	0.0000	0.0131156	58.37
HySimODE	5.1036	5.2620	2528.79	136342.30	0.0247845	115.18

4.2 Distributional comparison

Figures 2 and 3 show the final-time distributions of M and P obtained from 200 trajectories. SSA serves as the reference solution. HySimODE closely reproduces the SSA distributions

for both species, matching their means, variances, and overall support. Its accuracy is comparable to that of COPASI Hybrid, with minor deviations attributable to the deterministic treatment of selected reaction channels in hybrid schemes. As expected, the protein (P) distribution exhibits substantially greater spread due to noise amplification during translation, a feature captured consistently by HySimODE.

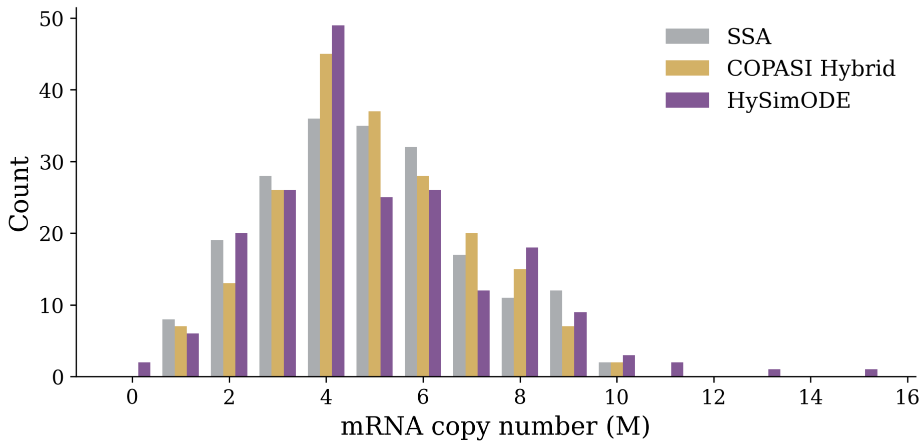

Figure 2: Final-time distribution of mRNA (M) at $t = 200$ obtained from SSA, COPASI Hybrid, and HySimODE using 200 independent trajectories. All three methods yield comparable distributions in both shape and support. Small deviations between SSA and the hybrid approaches are expected due to their partially deterministic treatment of selected reaction channels.

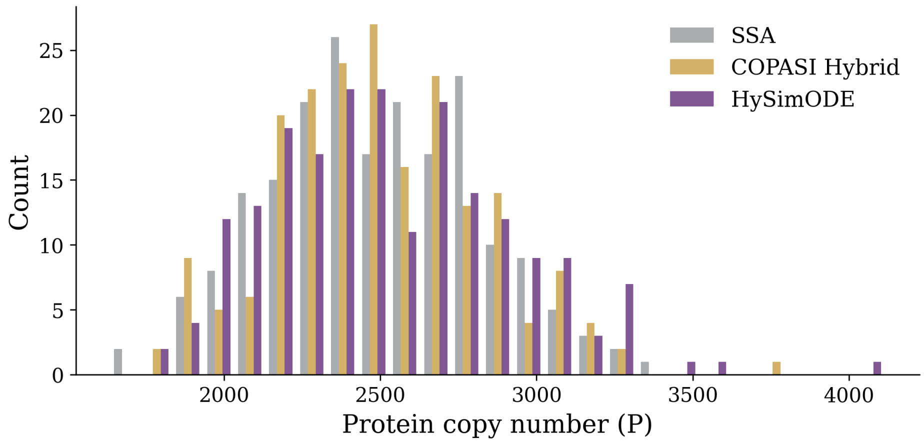

Figure 3: Final-time distribution of protein (P) at $t = 200$ for SSA, COPASI Hybrid, and HySimODE. Protein displays larger variance due to noise amplification during translation. Both hybrid methods reproduce the overall shape and range of the SSA distribution while retaining expected differences inherent to hybrid stochastic–deterministic formulations.

4.3 Mean trajectory comparison

Figures 4 and 5 show the time evolution of the mean mRNA (M) and protein (P) copy numbers computed from 200 independent trajectories. All stochastic and hybrid methods follow the deterministic ODE solution closely after the initial transient and converge to the same steady-state values. HySimODE reproduces both the transient and steady-state behaviour with high fidelity, matching the SSA and COPASI Hybrid means throughout the simulation window and accurately capturing the early-time stochastic relaxation dynamics.

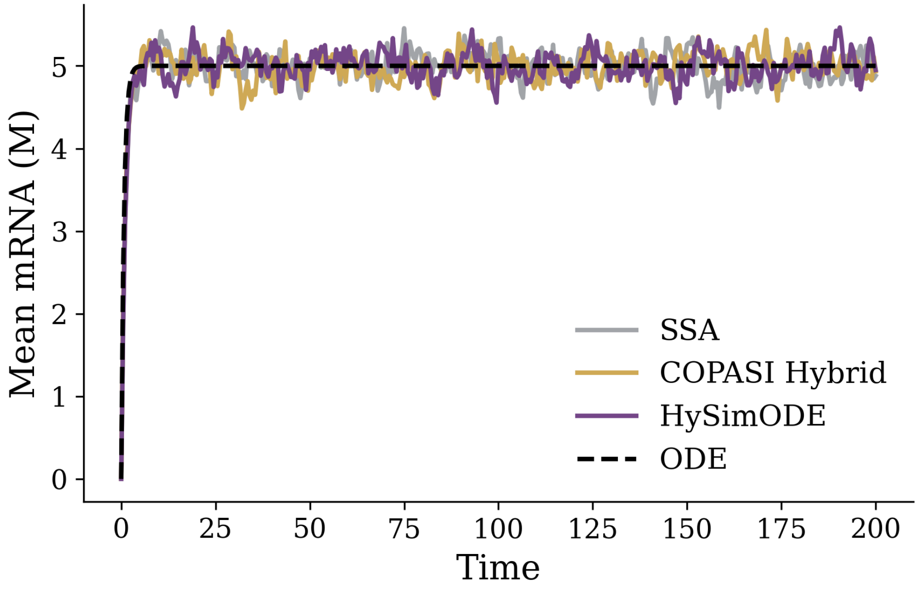

Figure 4: Time evolution of the mean mRNA copy number (M) computed from 200 trajectories using SSA, COPASI Hybrid, and HySimODE, together with the deterministic ODE solution. All stochastic methods converge to the same steady-state mean and closely follow the deterministic trajectory after the initial transient, demonstrating accuracy and numerical stability of the hybrid solvers.

4.4 Summary

Overall, the benchmark shows that HySimODE reproduces SSA-level mean and variance behaviour while avoiding the need for an explicit reaction-network reconstruction. HySimODE also matches the SSA and COPASI Hybrid variance trajectories throughout the simulation window, indicating that the method accurately captures noise propagation over time. Runtime measurements further show that HySimODE provides a favourable accuracyperformance trade-off, achieving distribution-level accuracy close to SSA while remaining substantially faster than fully stochastic methods. These properties make HySimODE suitable for multiscale biochemical models defined only at the ODE level, where full SSA is infeasible and reaction-network-based hybrid methods cannot be applied.

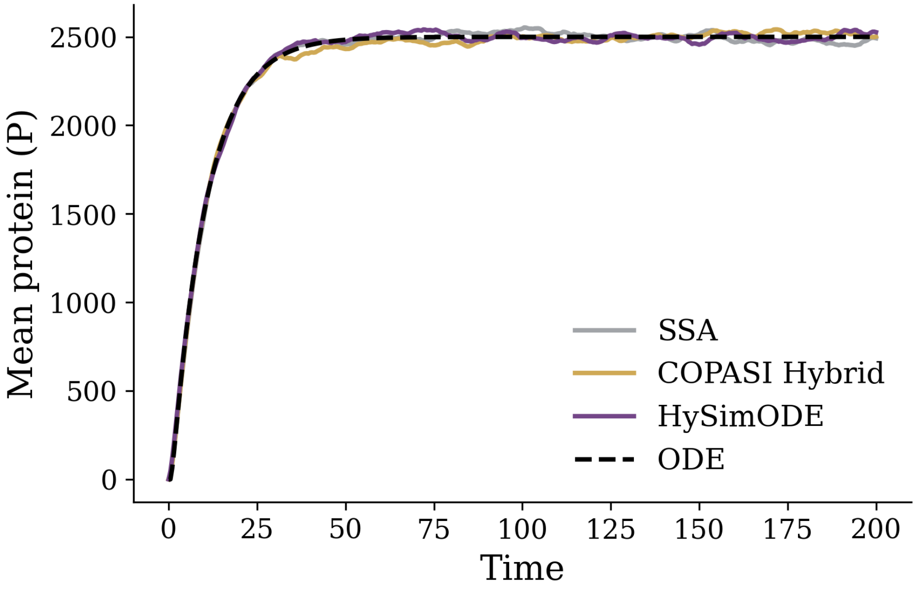

Figure 5: Time evolution of the mean protein copy number (P) for SSA, COPASI Hybrid, HySimODE, and the deterministic ODE solution. Hybrid methods accurately match the deterministic transient and steady-state behavior, with residual fluctuations originating from the stochastic components of the dynamics. The close agreement among all methods highlights the consistency and reliability of the hybrid simulation framework.

5 Testing of the hybrid simulator on unseen models

To demonstrate the performance of the hybrid simulator, we applied HySimODE to two nonlinear multiscale models with distinct structures and time scales:

1. the 31-equation hostrepressilator system, which couples resource-aware host physiology with a synthetic genetic oscillator; and

2. the 23-equation bicompartmental synaptic tagging cascade involved in synaptic plasticity.

These case studies differ markedly in topology, copy-number regimes, and characteristic times, providing a stringent benchmark for hybrid performance. For both systems, the corresponding binary regime labels (`0` = deterministic, `1` = stochastic) are stored in `output_hybrid/rfc_decisions.csv`, automatically generated by `hysimode.py`. Together, the trajectory visualizations stored in `output_hybrid/` and label tables provide a consistent validation that the RFC partition aligns with both biological intuition and the abundance-based thresholds used during training.

5.1 Hybrid simulation of the host–repressilator model

To evaluate whether the hybrid stochastic-deterministic simulator (HySimODE) preserves both the qualitative and quantitative dynamics of representative species in the host-repressilator system, we analyzed multiple independent hybrid realizations for a low-copy molecular component. Specifically, we focused on the ribosomemRNA complex `rmg1`, which participates in the translation of the first repressilator gene (`g1`). This complex reflects a biologically relevant stochastic node, as it directly links gene expression to host resource allocation. Three independent hybrid runs were performed (`results_run1.csv`, `results_run2.csv`, `results_run3.csv`) under identical model parameters but with different random seeds, all sharing the same temporal grid (`time.txt`). Each realization introduces intrinsic noise only in the RFC-classified stochastic subset of variables, while deterministic species are integrated continuously. Post-processing and ensemble statistics (mean and standard deviation) were computed automatically using `src/analyze_runs.py` and stored in the output directory (`output_hybrid/runs/`).

Figure 6a shows the resulting hybrid stochastic trajectories for `rmg1`. Individual realizations (blue tones) exhibit small fluctuations in amplitude and phase yet remain synchronized within the same oscillatory envelope, indicating that the hybrid scheme preserves the repressilators limit-cycle attractor, consistent with the oscillatory behavior described by Weisse et al. [Weiße et al., 2015].

The ensemble mean (black line) captures the deterministic backbone of the oscillation, while the variability across runs reflects the intrinsic stochasticity expected in low-copy translation intermediates. These results demonstrate that HySimODE maintains global dynamical consistency while introducing biologically plausible molecular noise, validating both the classifier-based partition and the hybrid stochastic update strategy.

In addition, we visualized 25 biochemical variables of the `host_repressilator` model (Fig-

ure 7). Each subplot displays the hybrid trajectory of one species, color-coded according to its RFC-assigned regime: deterministic species (sky blue) and stochastic species (gray). This visualization emphasizes the classifiers ability to distinguish between high-abundance, stable components (e.g., ribosomal and metabolic intermediates) and low-copy, noise-dominated mRNAs.

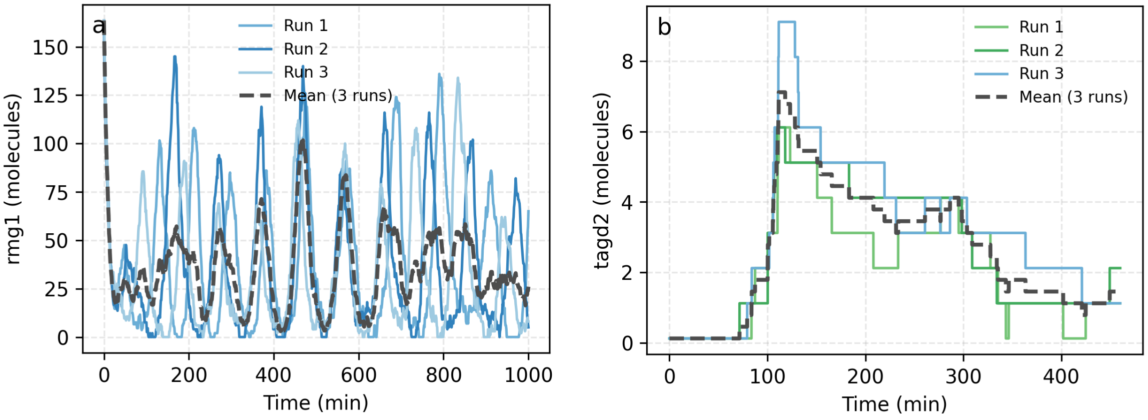

Figure 6: **Hybrid stochastic trajectories for representative species in the hostrepressilator and Smolen models.** (a) Stochastic trajectories of the ribosomemRNA complex `rmg1` from the hostrepressilator system across three independent hybrid simulation runs (blue tones), together with their mean trajectory (black line). The traces illustrate the intrinsic variability captured by the stochastic component while preserving the global oscillatory envelope characteristic of the deterministic attractor. (b) Stochastic trajectories of `tagd2` from the Smolen synaptic tagging model across three hybrid runs (purple tones) with the corresponding mean trajectory (black). The results show discrete jump-like dynamics typical of low-copy-number molecular species and demonstrate that **HySimODE** reproduces consistent ensemble averages while capturing stochastic fluctuations inherent to signaling intermediates.

5.2 Hybrid simulation of the Smolen model

To further evaluate the hybrid simulator under a biologically realistic signaling context, we applied it to the 23-species synaptic tagging and long-term potentiation model of Smolen et al. [Smolen et al., 2012]. In this system, transient kinase activation (e.g., ERK, CaMKII) drives the synthesis and decay of synaptic tags that determine the transition from early to late long-term potentiation. Figure 8 shows trajectories for 20 representative species obtained under hybrid simulation. Species classified as deterministic by the RFC are shown in blue, while those assigned to the stochastic regime appear in gray. The resulting partition reflects the heterogeneous nature of the signaling cascade: abundant components such as `Raf_d`, `psit1`, and `PRP` are consistently deterministic, whereas low-copy intermediates (e.g., `tagd1`, `tagd2`, `MEKpp_s`) are classified as stochastic. This separation aligns with biological expectations that high-abundance proteins buffer intrinsic noise, while low-copy signaling elements remain fluctuation-sensitive.

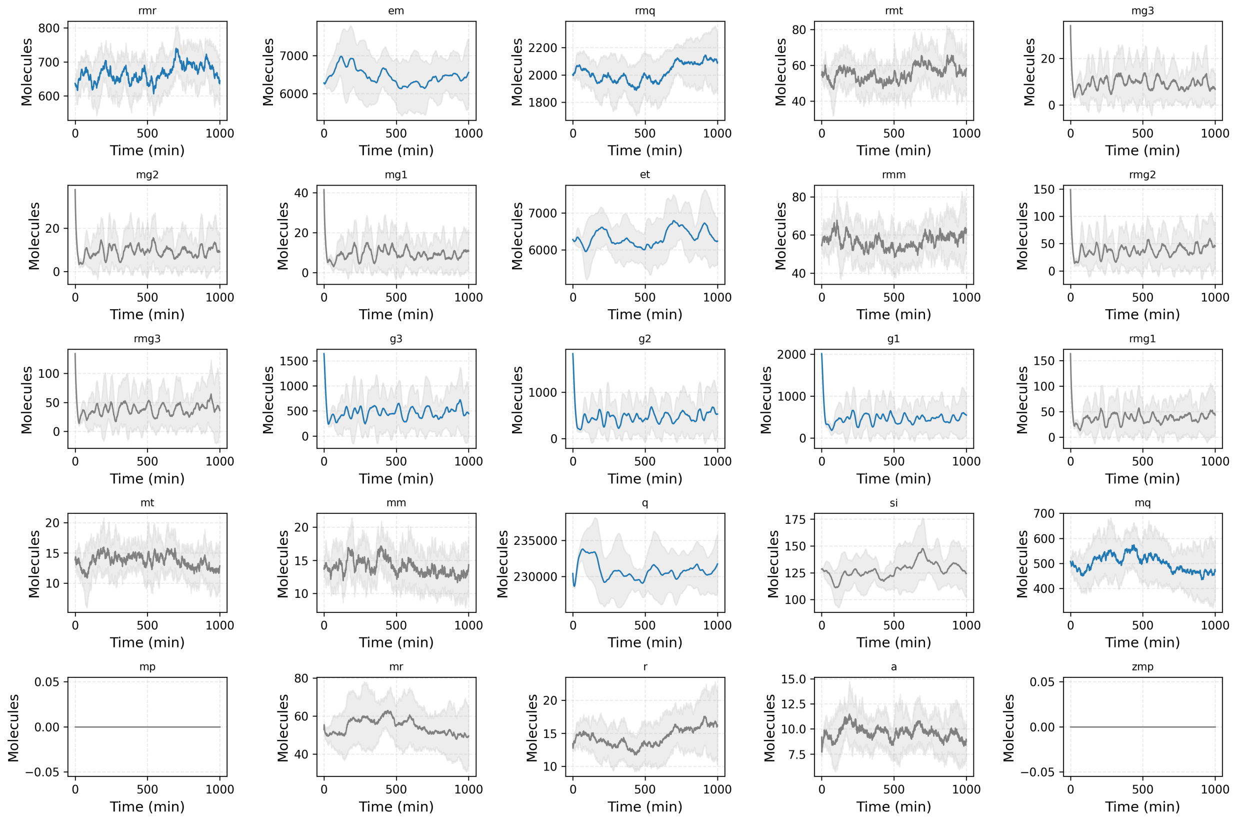

Figure 7: **Hybrid simulation of the host–repressilator model.** Trajectories of 25 repressentative biochemical species from the hostrepressilator system [Weiße et al., 2015]. Species classified as deterministic by the RFC are shown in sky blue, while those classified as stochastic are shown in gray. Deterministic variables such as `rmr`, `em`, `rmq`, `et`, and `g1-g3` exhibit smooth, continuous trajectories, whereas low-copy species sucha as `mg1-mg3`, `rmg1`, `mt`, `mr`, `r` and `a` display noisy fluctuations. This hybrid simulations demonstrates how HySimODE partitions and integrates a complex system linking host physiology and a synthetic oscillator, reproducing deterministic and stochastic behaviors.

The hybrid trajectories of `tagd2` shown in Figure 6b further illustrate intrinsic noise behavior, revealing stochastic fluctuations superimposed on a reproducible transient response. Among these intermediates, `tagd2` represents a low-abundance signaling tag whose concentration fluctuates due to upstream stochastic activation of kinases and limited molecular copy number.

Furthermore, twenty independent hybrid realizations were generated under identical parameters but different random seeds, and their ensemble statistics were computed using `analyze_runs.py`. Across all runs, `tagd2` exhibits irregular temporal fluctuations superimposed on a reproducible mean decay profile, consistent with the transient tagging phase predicted by deterministic dynamics. The gray envelope in Figure 8 highlights the variability region (±1 SD), capturing intrinsic stochastic dispersion while preserving the characteristic activationdecay trajectory of the model.

Together, these examples confirm that **HySimODE** generalizes across distinct biochemical regimesoscillatory genetic circuits and transient signaling cascadespreserving dynamical fidelity while efficiently capturing intrinsic noise in low-copy molecular species. In addition, validation on a minimal two-compartment gene-expression model demonstrates that the concentration-to-molecule adapter operates generically for compartmental ODE systems defined in concentration units, without requiring model-specific assumptions.

5.3 Validation of the concentration-to-molecule adapter using a generic two-compartment gene expression model

To demonstrate the general applicability of the concentration-to-molecule adapter, we constructed a minimal two-compartment gene expression model (Figure 9) representing nuclear transcription, mRNA export to the cytosol, and protein translation. The model consists of two compartments: a nuclear compartment where transcription occurs and a cytosolic compartment where translation takes place. The nuclear species corresponds to nuclear mRNA (`mRNA_nuc`), while cytosolic species include exported mRNA (`mRNA_cyt`) and the translated protein (`Protein`). mRNA produced in the nucleus is exported to the cytosol, where it is translated into protein and eventually degraded.

The model is defined entirely in concentration units (μM) and specifies compartment structure through the generic interfaces `compartment_indices` and `compartment_volumes_L`. Using the concentration adapter, HySimODE automatically converts the concentration-based dynamics into molecule units prior to simulation.

HySimODE then performs the deterministic pre-simulation, RFC-based species classification, and hybrid stochasticdeterministic simulation without any model-specific assumptions. This example confirms that the adapter operates generically for compartmental ODE systems and is not tied to any particular biological model structure.

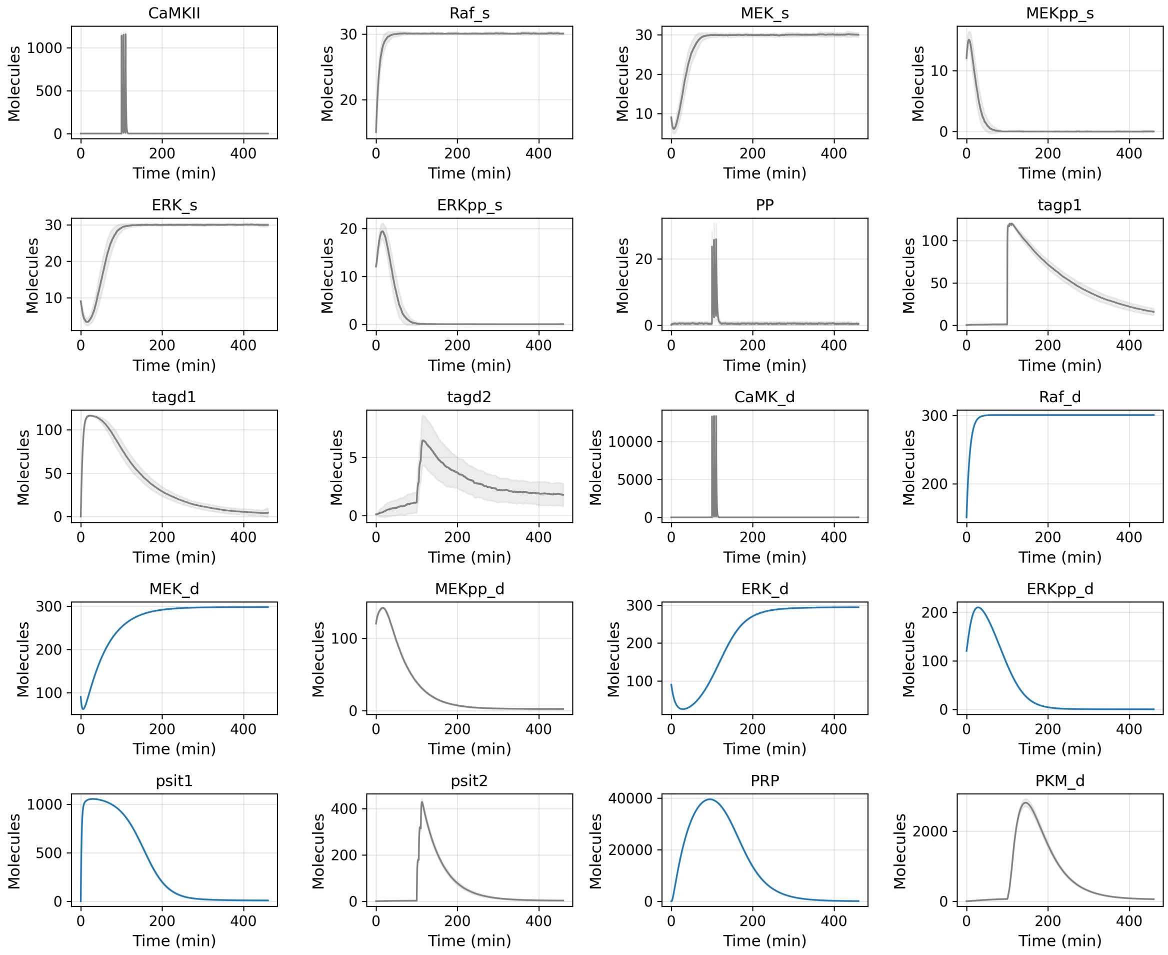

Figure 8: **Hybrid dynamics of the Smolen synaptic tagging model.** The figure shows the hybrid mean (solid line) and standard deviation (gray shading) computed over 20 independent hybrid realizations for 20 representative species. Stochastic variables, including `tagd2`, exhibit higher amplitude variability while maintaining consistent mean trajectories. The panel for `tagd2` (second row, third column) illustrates the expected transient activation and decay characteristic of the tagging process, with biologically plausible fluctuations emerging from the hybrid stochastic update. The partition aligns with biological intuition: abundant proteins (e.g., `PRP`, `psit1`, `Raf_d`) behave deterministically, while low-copy intermediates (`tagd1`, `tagd2`, `MEKpp_s`) exhibit stochastic fluctuations.

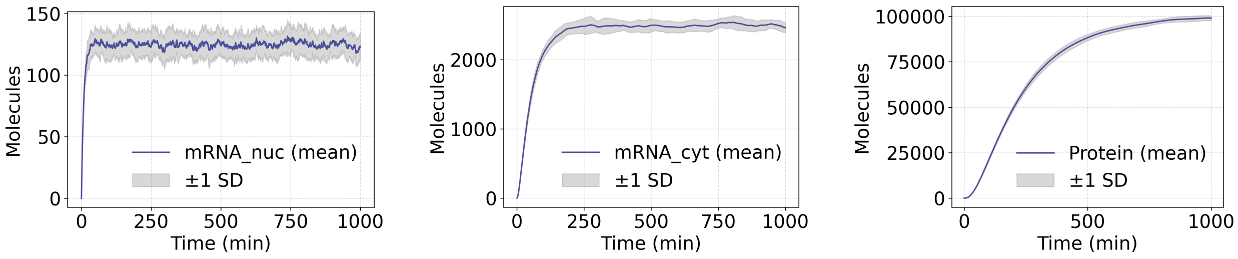

(a) `mRNA_nuc` (b) `mRNA_cyt` (c) `Protein`

Figure 9: **Validation of the concentration-to-molecule adapter using a generic two-compartment gene expression model.** The model is defined in concentration units (μM) with nuclear and cytosolic compartments and is converted to molecule counts via the concentration adapter prior to hybrid simulation. Solid lines show the mean trajectory across runs and shaded regions indicate ± 1 SD. The nuclear mRNA exhibits larger relative fluctuations due to lower copy number, while cytosolic mRNA and protein approach high-copy steady states with smaller relative noise.

6 User guide

HySimODE is a Python framework for hybrid stochasticdeterministic simulation of biochemical networks. It integrates a **Random Forest Classifier (RFC)** that automatically assigns each species to a simulation regime (stochastic or deterministic), and a unified hybrid integrator that couples ODE and SSA dynamics on a shared time grid (`dt`). The following subsections describe installation, model configuration, simulation execution, and result analysis to reproduce all simulations reported in this study.

6.1 Installation and dependencies

- **Python version:** 3.12.3
- **Required packages:** `numpy`, `pandas`, `scipy`, `matplotlib`, `scikit-learn`

To install and run **HySimODE**:

```
git clone https://github.com/criseidazamora/HySimODE.git
cd HySimODE
pip install -r requirements.txt
```

6.2 Model setup

Each biochemical model must be defined as a self-contained Python file containing the following elements:

- `odes(t, y, params)` the system of ODEs.
- `params` a dictionary of model parameters.
- `Y0` a vector of initial conditions.
- `var_names` an ordered list of species names.

Two example models distributed with the repository are:

- `host_repressilator.py` 31 species, adapted from Weiße et al. [2015].
- `smolen_odes.py` 23 species, adapted from Smolen et al. [2012].

An illustrative example, `my_ode_model.py`, is included in the repository under `models/`. This toy model demonstrates the minimal structure required by the simulator:

6.3 Running hybrid simulations

The main entry point is `hysimode.py`. The simulator automatically:

1. Loads the ODE model and its configuration.
2. Computes quantitative features for each species.
3. Applies the trained RFC to assign regime labels (`0 = deterministic`, `1 = stochastic`).

4. Runs the hybrid integrator, coupling ODE and SSA dynamics.
5. Saves trajectories and classification results in `output_hybrid/`.

Example 1 molecule-based model:

```
python hysimode.py --model host_repressilator.py \
--tfinal 1000 --dt 1.0 --runs 3
```

Example 2 concentration-based model (via concentration-to-molecule number adapter):

```
BASE_MODEL=smolen_odes python hysimode.py \
  --model concentration_adapter_hybrid.py --tfinal 460 --dt 0.01 --runs 3
```

The variable `BASE_MODEL` specifies which concentration-based model will be loaded through the generic adapter `concentration_adapter_hybrid.py`, which automatically converts between M and molecules as units.

Note (Windows users): In PowerShell or CMD, the environment variable must be set in a separate command:

```
set BASE_MODEL=smolen_odes
python hysimode.py --model concentration_adapter_hybrid.py \
--tfinal 460 --dt 0.01 --runs 3
```

This replaces the Unix-style syntax `BASE_MODEL=smolen_odespython...`.

6.3.1 Concentration-to-molecule adapter

Many biochemical models are formulated as systems of ordinary differential equations (ODEs) in concentration units (typically μM), whereas stochastic simulation algorithms operate on discrete molecule counts. To enable the direct use of concentration-based models in hybrid simulations, HySimODE includes a concentration-to-molecule adapter.

The adapter automatically converts state variables between concentration and molecule units using

$$n = c \cdot 10^{-6} \cdot V \cdot N_A,$$

where c is the concentration in μM, V is the compartment volume in liters, and N_A is Avogadro's number.

The adapter supports arbitrary multi-compartment models through a general interface in which models define species-to-compartment mappings and compartment volumes. This design avoids model-specific assumptions and allows existing ODE models to be used directly within the hybrid simulation framework without modification.

The accuracy of the hybrid solver depends on the synchronization interval Δt used to couple the deterministic and stochastic components. Smaller values of Δt lead to more accurate ODE–SSA coupling, as propensities are updated more frequently, although this increases

computational cost. In practice, values of Δt in the range 0.01 to 0.05 provided stable results for the models considered here.

The adapter acts as a wrapper around the original ODE model and does not modify the model equations themselves.

6.4 Post-processing and visualization

Results from multiple hybrid runs can be analyzed using `analyze_runs.py`. This script computes ensemble means and standard deviations for all species and optionally produces plots:

```
python analyze_runs.py --dir output_hybrid/runs --plot
```

Mean trajectories (1 SD) are saved under: `output_hybrid/summary/plots/`.

6.5 Output interpretation

The directory structure after a simulation is as follows:

- `output_hybrid/runs/` raw trajectories from all hybrid runs.
- `output_hybrid/rfc_decisions.csv` RFC-based regime assignments for each species.
- `output_hybrid/summary/species_summary.csv` mean and SD across runs.
- `output_hybrid/summary/plots/*.png` visualization of mean SD trajectories.
- `output_hybrid/timeseries_txt/` plain-text time series of the last run.
- `output_hybrid/trajectory_*.png` — plots of individual trajectories from the last run

6.6 Extending HySimODE to new models

To apply **HySimODE** to a new system, define a model file `my_model.py` containing: `odes`, `params`, `Y0`, and `var_names`. Place it in the same directory as `hysimode.py` and ensure the RFC artifacts (`rfc_integration.py`, `rfc_calibrated.joblib`, and `rfc_metadata.json`) are present. Note: the illustrative example, `my_ode_model.py`, is included in the repository under `models/`.

Run the hybrid simulator as follows:

```
python hysimode.py --model my_model.py --tfinal 2000 --dt 1.0 --runs 3
```

The framework automatically adapts to the dimensionality and scaling of any new model, allowing plug-and-play use without manual tuning.

Tutorials and case studies are available in the `case_studies/` folder. Representative simulation outputs are also included in the repository; these correspond to reduced numbers of runs and shorter simulation times for efficiency. The figures reported in the manuscript can be fully reproduced by increasing the number of runs and simulation horizon accordingly.

6.7 Main output files

Output directory. All simulation outputs are automatically stored under the `output_hybrid/` directory. The folder is created during each hybrid simulation run and contains the following files and subfolders:

- `output_hybrid/runs/results_runX.csv` — Time-series data for each hybrid run ($X = 1, 2, \ldots$).
- `output_hybrid/rfc_decisions.csv` — RFC-predicted labels for each species (0 = deterministic, 1 = stochastic).
- `output_hybrid/summary/species_summary.csv` — Mean and standard deviation across runs, generated automatically by `analyze_runs.py`.
- `output_hybrid/summary/plots/*.png` — Mean ± SD trajectories for all species.
- `output_hybrid/timeseries_txt/*.txt` — Plain-text time series from the last simulation run.

These outputs provide a complete record of simulation results, classification decisions, and ensemble statistics for reproducibility and downstream analysis.

7 Code and Model Availability

The trained Random Forest Classifier (RFC) and the **HySimODE** Python framework are freely available at `https://github.com/criseidazamora/HySimODE`. The repository contains all source code, model files, and training datasets required to reproduce the results presented in this work.

7.1 Training model collection

The RFC was trained using features extracted from a curated and systematically constructed collection of ODE models. The dataset combines canonical models from the literature with synthetic systems and systematically generated variants based on common biochemical kinetic principles (e.g., mass-action, Michaelis–Menten, and regulatory feedback mechanisms). Canonical models from the literature (e.g., genetic circuits such as the repressilator Elowitz and Leibler [2000] and toggle switch Gardner et al. [2000], network motifs such as the incoherent feed-forward loop Mangan and Alon [2003], and classical nonlinear oscillators including the Brusselator Prigogine and Lefever [1968], Oregonator Field and Noyes [1974], FitzHugh–Nagumo model FitzHugh [1961], Nagumo et al. [1962], and Goodwin oscillator Goodwin [1965]) were included alongside synthetic variants constructed in this study.

Starting from a set of representative base models, additional variants were designed through structural modifications and parameterizations to ensure diversity across dynamical regimes and to avoid trivial classification patterns. The resulting training set comprises the following model families:

- Core Metabolism / Smooth Dynamics (Deterministic ODEs):

1. `metab01_linear_pathway.py`
   Linear metabolic chain with mass-action kinetics.
2. `metab02_linear_pathway_MM.py`
   Same as metab01, with MichaelisMenten saturation in one step.
3. `metab03_linear_pathway_feedback.py`
   Linear pathway with negative feedback inhibition (Hill-type).
4. `metab04_reduced_glycolysis.py`
   Reduced glycolysis-like system (existing A3-type model).
5. `metab05_glycolysis_intermediate.py`
   Glycolysis variant with explicit intermediate buffering species.
6. `metab06_glycolysis_pulsed_input.py`
   Glycolysis variant with time-dependent input flux.

- Phosphorylation & Signaling Cascades:
  1. `signal01_mapk_single_layer.py`
     Single-layer MAPK phosphorylationdephosphorylation cycle.
  2. `signal02_mapk_double_phosphorylation.py`
     MAPK → MAPKp → MAPKpp cascade.
  3. `signal03_mapk_with_phosphatase.py`
     MAPK cascade with explicit phosphatase enzyme.
  4. `signal04_goldbeter_koshland.py`
     GoldbeterKoshland ultrasensitive switch.
  5. `signal05_mapk_three_layer.py`
     Three-layer MAPK cascade (RafMEKERK simplified).
  6. `signal06_mapk_three_layer_feedback.py`
     Same as signal05, with ERK negative feedback on upstream kinase.
- ReceptorLigand Binding and Trafficking:
  1. `rl01_ligand_receptor_binding.py`
     Ligandreceptor association/dissociation.
  2. `rl02_receptor_activation.py`
     Activated receptor state (LR → R*).
  3. `rl03_receptor_internalization.py`
     Internalization and recycling of activated receptor.
  4. `rl04_ligand_degradation.py`
     Ligand turnover with production and degradation.
- Deterministic Oscillators:

1. `osc01_lotka_volterra.py`
   Classical predatorprey system.

2. `osc02_lotka_volterra_logistic.py`
   Predatorprey with carrying capacity.

3. `osc03_goodwin_oscillator.py`
   Goodwin-type negative feedback oscillator.

4. `osc04_goodwin_delay_chain.py`
   Goodwin oscillator with delay approximated by intermediate chain.

- Gene Regulatory Circuits (Low Copy / Noise-Prone):

  1. `grn01_repressilator.py`
     Canonical repressilator circuit.

  2. `grn02_repressilator_saturable_deg.py`
     Repressilator with saturable protein degradation.

  3. `grn03_repressilator_dimerization.py`
     Repressilator with dimeric repressors.

  4. `grn04_toggle_switch.py`
     Canonical genetic toggle switch.

  5. `grn05_toggle_autoregulation.py`
     Toggle switch with additional autoregulatory loop.

  6. `grn06_toggle_asymmetric.py`
     Toggle switch with asymmetric production rates.

  7. `grn07_lambda_switch.py`
     Lambda phagetype bistable switch.

  8. `grn08_lambda_switch_cooperative.py`
     Lambda switch with higher-order cooperativity.

- Network Motifs / Multiscale Models:

  1. `motif01_i1ffl_incoherent.py`
     Incoherent type-1 feed-forward loop.

  2. `motif02_i1ffl_coherent.py`
     Coherent feed-forward loop.

  3. `motif03_i1ffl_saturable_output.py`
     I1FFL with saturable output node.

  4. `motif04_stochastic_gene_deterministic_protein.py`
     Hybrid model: stochastic gene expression controlling deterministic protein dynamics.

5. motif05_stochastic_enzyme_deterministic_substrate.py
   Hybrid model: low-copy enzyme with abundant substrate.

6. motif06_delayed_negative_feedback_pulse.py
   Delayed negative-feedback pulse generator.

- Noise-Prone Oscillatory Models:

  1. stochosc01_brusselator.py
     Two-species Brusselator chemical oscillator exhibiting nonlinear limit-cycle dynamics.

  2. stochosc02_oregonator_reduced.py
     Reduced three-species Oregonator model of the BZ reaction.

  3. stochosc03_activator_inhibitor.py
     Activatorinhibitor oscillator with positive feedback and delayed inhibition.

  4. stochosc04_fitzhugh_nagumo_like.py
     FitzHughNagumolike excitable oscillator adapted to biochemical kinetics.

  5. stochosc05_brusselator_saturable_deg.py
     Brusselator variant with saturable degradation kinetics.

  6. stochosc06_activator_inhibitor_pulsed_input.py
     Activatorinhibitor oscillator driven by a time-dependent pulsed input.

7.2 Core scripts of the computational framework

HySimODE is composed of the following core scripts:

1. `hysimode.py` — Executes hybrid deterministicstochastic simulations using the RFC-derived classification.

2. `rfc_integration.py` — Provides auxiliary functions to embed the RFC within the hybrid simulator. This script loads the files `rfc_metadata.json` and `rfc_calibrated.joblib`, both generated by the training script `train_rfc.py`.

3. `concentration_adapter_hybrid.py` — Automatically converts concentrations to molecule numbers. This script is required because the RFC classifier was trained on molecule counts.

7.3 Training the RFC

The following scripts are used to train the Random Forest Classifier (RFC):

1. `make_features_rfc.py` — Simulates the training models and extracts features into a CSV file.

2. `train_rfc.py` — Trains the RFC and saves the classifier as a `.joblib` file with associated metadata (`.json`).

Output folder. All intermediate and final results produced during RFC training are stored under the automatically created `outputs/` directory inside each corresponding pipeline folder. These outputs include:

- Feature matrices used for training (`features_rfc.csv`)
- Final trained model files (`rfc_calibrated.joblib`, `rfc_metadata.json`)

7.4 Testing the RFC on unseen models

Independent testing and label prediction on models not used for training are performed with:

1. `make_features_host_repressilator.py` and `predict_with_rfc_on_host_repressilator.py`
2. `make_features_smolen.py` and `predict_with_rfc_on_smolen.py`

For concentration-based models (e.g., `smolen_odes.py`), features are computed on molecule units by running the model through `concentration_adapter_hybrid.py` (M $\leftrightarrow$ molecules conversion), ensuring consistency with the training distribution.

Output folder. Each testing pipeline automatically creates an `outputs/` directory that stores:

- Feature tables extracted from the ODE trajectories (`features_host_repressilator.csv` and `features_smolen.csv`)
- Predicted RFC labels for each species (`rfc_predictions.csv`) and probability scores and metadata used in downstream hybrid simulations

All results remain localized within each test models folder for clarity and reproducibility.

7.5 Testing models used in this study

Unseen models employed for hybrid simulations (`hysimode.py`) in this study were:

- `host_repressilator.py` — 31-variable host–circuit model (resource allocation + repressilator).
- `smolen_odes.py` — 23-variable synaptic tagging and capture model under pulsed kinase activation.

These models are provided in the `hysimode/models/` directory of the public repository.

Note. The adapter `concentration_adapter_hybrid.py` is used during hybrid simulations (`hysimode.py`) of concentration-defined models, but it is not used for feature generation in this work.

In addition, the gene-expression benchmark model used for the statistical comparison in Table 9 is provided in `hysimode/models/gene_expression.py`. This implementation includes

both the standard ODE formulation and the optional productiondegradation decomposition via `odes_prod_deg`, illustrating the extended HySimODE model interface.

7.6 Model library auditing and RFC sensitivity analysis

Additional scripts were developed to validate the diversity of the training model library and to evaluate the robustness of the Random Forest Classifier (RFC) used in HySimODE.

Model library audit. To ensure that the training models covered a broad range of dynamical behaviors, the scripts `audit_model.py` and `audit_model_similarity.py` were used to analyze the structural and dynamical diversity of the ODE model collection. These scripts compute feature-based similarity metrics between models and identify potential redundancies within the training set.

RFC threshold sensitivity. The script `threshold_sensitivity_rfc.py` evaluates the sensitivity of the RFC predictions to the classification threshold used to distinguish stochastic and deterministic regimes. This analysis assesses the robustness of the classifier and confirms that the hybrid regime predictions remain stable across a range of threshold values.

These scripts are provided for transparency and methodological validation but are not required for running hybrid simulations with HySimODE.

7.7 Reproducibility

All scripts are implemented in Python (3.12.3) and rely on standard scientific libraries (`NumPy`, `SciPy`, `pandas`, `matplotlib`, `scikit-learn`). Each script is designed to run independently, and together they form a reproducible pipeline from feature generation and RFC training to hybrid simulation and analysis.

Computing environments and platforms

All runs were reproduced on Windows (laptop and desktop), using consistent commands and the same repository snapshot.

Operating systems.

- **Laptop:** Windows 11 (tested via PowerShell and Command Prompt).
- **Desktop:** Windows 10 (tested via PowerShell and Command Prompt).

Python interpreters.

- **Primary:** Python 3.12.3 (Windows 10/11 and WSL). All experiments and figures for the manuscript were run under this version.
- **Compatibility test:** Python 3.10 (Windows). To ensure cross-version compatibility, we pinned Windows-compatible wheels:

– `numpy 1.26.4`, `scipy 1.11.4`, `pandas 2.2.2`, `matplotlib 3.8.4`, `scikit-learn 1.5.1`, `joblib 1.4.2`.

Shells and invocation notes.

- **Windows (PowerShell/CMD):**

```
set BASE_MODEL=smolen_odes
python hysimode.py --model concentration_adapter_hybrid.py --tfinal
    500 --dt 0.5 --runs 3
```

- **Linux/WSL/macOS (bash):**

```
BASE_MODEL=smolen_odes python hysimode.py --model
    concentration_adapter_hybrid.py --tfinal 500 --dt 0.5 --runs 3
```

System configuration and validation. All hybrid simulations were successfully executed on both *Windows* (10 and 11) and *Linux* (Ubuntu 24.04) systems. Tests were performed on a desktop workstation (*Intel* i9–14900, 128 GB RAM, *Windows* 10) and a laptop (*Intel* i7–10510U, 16 GB RAM, *Windows* 11). **HySimODE** ran consistently under Python 3.12.3 across all environments, with additional validation under Python 3.10 confirming cross-version compatibility after pinning the appropriate dependency versions.

Control of BLAS Threading For numerical stability on multi-core systems, the number of BLAS threads used by NumPy/SciPy was restricted to one. High thread counts in OpenBLAS or MKL may occasionally lead to instability during long ODE integrations. Therefore, the following environment variables were set during all simulations:

```
OMP_NUM_THREADS=1
OPENBLAS_NUM_THREADS=1
MKL_NUM_THREADS=1
NUMEXPR_NUM_THREADS=1
```

Together, the public repository, training model library, and documented execution environment provide a reproducible computational workflow for RFC training, hybrid regime prediction, and hybrid simulation with HySimODE.

References

David F. Anderson. A modified next reaction method for simulating chemical systems with time dependent propensities and delays. *The Journal of Chemical Physics*, 127(21):214107, 2007.

Leo Breiman. Random forests. *Machine Learning*, 45(1):5–32, 2001.

Yang Cao, Daniel T. Gillespie, and Linda R. Petzold. Avoiding negative populations in explicit poisson tau-leaping. *The Journal of Chemical Physics*, 123(5):054104, 2005.

Johan Elf and Måns Ehrenberg. Spontaneous separation of bi-stable biochemical systems into spatial domains of opposite phases. *Systems Biology*, 1(2):230–236, 2003.

Michael B. Elowitz and Stanislas Leibler. A synthetic oscillatory network of transcriptional regulators. *Nature*, 403(6767):335–338, 2000.

Michael B. Elowitz, Eric J. Levine, Eric D. Siggia, and Peter S. Swain. Stochastic gene expression in a single cell. *Science*, 297(5584):1183–1186, 2002.

Richard J Field and Richard M Noyes. Oscillations in chemical systems. iv. limit cycle behavior in a model of a real chemical reaction. *The Journal of Chemical Physics*, 60(5): 1877–1884, 1974.

Richard FitzHugh. Impulses and physiological states in theoretical models of nerve membrane. *Biophysical Journal*, 1(6):445–466, 1961.

Timothy S. Gardner, Charles R. Cantor, and James J. Collins. Construction of a genetic toggle switch in *Escherichia coli*. *Nature*, 403(6767):339–342, 2000.

Daniel T. Gillespie. Exact stochastic simulation of coupled chemical reactions. *The Journal of Physical Chemistry*, 81(25):2340–2361, 1977.

Daniel T. Gillespie. The chemical langevin equation. *The Journal of Chemical Physics*, 113 (1):297–306, 2000.

Brian C Goodwin. Oscillatory behavior in enzymatic control processes. *Advances in Enzyme Regulation*, 3:425–438, 1965.

Eric L. Haseltine and James B. Rawlings. Approximate simulation of coupled fast and slow reactions for stochastic chemical kinetics. *The Journal of Chemical Physics*, 117(15): 6959–6969, 2002.

Stefan Hoops, Sven Sahle, Ralph Gauges, Christine Lee, Jürgen Pahle, Natalia Simus, Maneesh Singhal, Liang Xu, Pedro Mendes, and Ursula Kummer. Copasia complex pathway simulator. *Bioinformatics*, 22(24):3067–3074, 2006.

Timo R Maarleveld, Brett G Olivier, and Frank J Bruggeman. Stochpy: a comprehensive, user-friendly tool for simulating stochastic biological processes. *PLoS One*, 8(11):e79145, 2013.

Shmoolik Mangan and Uri Alon. Structure and function of the feed-forward loop network motif. *Proceedings of the National Academy of Sciences*, 100(21):11980–11985, 2003.

Harley H. McAdams and Adam Arkin. Stochastic mechanisms in gene expression. *Proceedings of the National Academy of Sciences*, 94(3):814–819, 1997.

J Nagumo, S Arimoto, and S Yoshizawa. An active pulse transmission line simulating nerve axon. *Proceedings of the IRE*, 50(10):2061–2070, 1962.

Johan Paulsson. Summing up the noise in gene networks. *Nature*, 427(6973):415–418, 2004.

Juan M. Pedraza and Alexander van Oudenaarden. Noise propagation in gene networks. *Science*, 307(5717):1965–1969, 2005.

Ilya Prigogine and René Lefever. Symmetry breaking instabilities in dissipative systems. ii. *The Journal of Chemical Physics*, 48(4):1695–1700, 1968.

Arjun Raj and Alexander van Oudenaarden. Nature, nurture, or chance: stochastic gene expression and its consequences. *Cell*, 135(2):216–226, 2008.

Björn Schwanhäusser, Dorothea Busse, Na Li, Gunnar Dittmar, Johannes Schuchhardt, Jana Wolf, Wei Chen, and Matthias Selbach. Global quantification of mammalian gene expression control. *Nature*, 473(7347):337–342, 2011.

Vahid Shahrezaei and Peter S. Swain. Analytical distributions for stochastic gene expression. *Proceedings of the National Academy of Sciences*, 105(45):17256–17261, 2008.

Paul Smolen, Douglas A. Baxter, and John H. Byrne. Molecular constraints on synaptic tagging and maintenance of long-term potentiation: A predictive model. *PLOS Computational Biology*, 8:1–17, 08 2012.

Peter S. Swain, Michael B. Elowitz, and Eric D. Siggia. Intrinsic and extrinsic contributions to stochasticity in gene expression. *Proceedings of the National Academy of Sciences*, 99 (20):12795–12800, 2002.

Yuichi Taniguchi, Paul J. Choi, Gene-Wei Li, Hui Chen, Mohan Babu, Jason Hearn, Andrew Emili, and Xiaoliang S. Xie. Quantifying e. coli proteome and transcriptome with single-molecule sensitivity in single cells. *Science*, 329(5991):533–538, 2010.

Mukund Thattai and Alexander van Oudenaarden. Intrinsic noise in gene regulatory networks. *Proceedings of the National Academy of Sciences*, 98(15):8614–8619, 2001.

N. G. van Kampen. *Stochastic Processes in Physics and Chemistry*. Elsevier, 3rd edition, 2007.

A. Y. Weiße, D. A. Oyarzún, V. Danos, and P. S. Swain. Mechanistic links between cellular trade-offs, gene expression, and growth. *Proceedings of the National Academy of Sciences of the United States of America*, 112(9):E1038–E1047, 2015.
